# In Vitro Simulated Neuronal Environmental Conditions Qualify Umbilical Cord Derived Highly Potent Stem Cells for Neuronal Differentiation

**DOI:** 10.1007/s12015-023-10538-w

**Published:** 2023-04-24

**Authors:** Jessika Maassen, Rebecca Guenther, Timm J. J. Hondrich, Bogdana Cepkenovic, Dominik Brinkmann, Vanessa Maybeck, Andreas Offenhäusser, Barbara Dittrich, Anna Müller, Claudia Skazik-Voogt, Maximilian Kosel, Christoph Baum, Angela Gutermuth

**Affiliations:** 1 Department for Applied Cell Biology, Fraunhofer Institute for Production Technology, Steinbachstr. 17, 52074 Aachen, Germany; 2 Institute for Biological Information Processing, IBI-3, Forschungszentrum Jülich GmbH, Leo Brandtstrasse Station 71, 52425 Jülich, Germany; 3 Department of Biology, RWTH Aachen University, Worringerweg 1, 52074 Aachen, Germany; 4 DWI-Leibniz Institute for Interactive Materials, Forckenbeckstrasse 50, 52074 Aachen, Germany

**Keywords:** Neuronal cell replacement, Tissue Engineering, Mechanotransduction, Adult mesenchymal stem cells, Umbilical cord tissue, Stem Cell differentiation, Stem cell spheroids, Wharton’s Jelly

## Abstract

The healing of neuronal injuries is still an unachieved goal. Medicine-based therapies can only extend the survival of patients, but not finally lead to a healing process. Currently, a variety of stem cell-based tissue engineering developments are the subject of many research projects to bridge this gap. As yet, neuronal differentiation of induced pluripotent stem cells (iPS), embryonic cell lines, or neuronal stem cells could be accomplished and produce functional neuronally differentiated cells. However, clinical application of cells from these sources is hampered by ethical considerations. To overcome these hurdles numerous studies investigated the potential of adult mesenchymal stem cells (MSCs) as a potential stem cell source. Adult MSCs have been approved as cellular therapeutical products due to their regenerative potential and immunomodulatory properties. Only a few of these studies could demonstrate the capacity to differentiate MSCs into active firing neuron like cells. With this study we investigated the potential of Wharton’s Jelly (WJ) derived stem cells and focused on the intrinsic pluripotent stem cell pool and their potential to differentiate into active neurons. With a comprehensive neuronal differentiation protocol comprised of mechanical and biochemical inductive cues, we investigated the capacity of spontaneously forming stem cell spheroids (SCS) from cultured WJ stromal cells in regard to their neuronal differentiation potential and compared them to undifferentiated spheroids or adherent MSCs. Spontaneously formed SCSs show pluripotent and neuroectodermal lineage markers, meeting the pre-condition for neuronal differentiation and contain a higher amount of cells which can be differentiated into cells whose functional phenotypes in calcium and voltage responsive electrical activity are similar to neurons. In conclusion we show that up-concentration of stem cells from WJ with pluripotent characteristics is a tool to generate neuronal cell replacement.

**Graphical Abstract:**

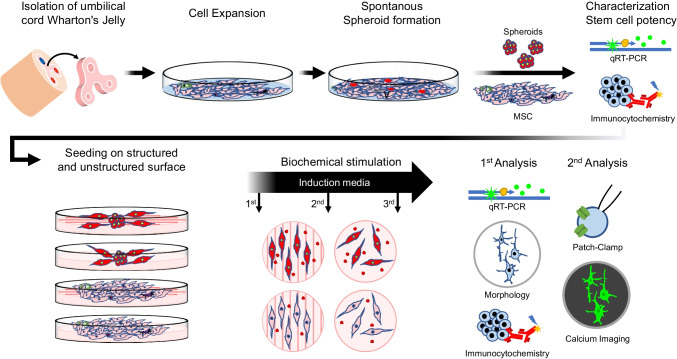

**Supplementary Information:**

The online version contains supplementary material available at 10.1007/s12015-023-10538-w.

## Introduction

Currently about 35 million people worldwide suffer from the incurable consequences of neurodegenerative diseases (NDD) and it is estimated that the number of cases will increase to 115 million by 2050 [1]. NDD are characterized by progressive dysfunction and loss of neuron function leading to mental, cognitive and / or motor disabilities and, ultimately, death [2]. One of the main reasons for such detrimental disease progression is the limited self-repair capacity of the mammalian peripheral and central nervous system. To date there is no effective treatment for NDD with the exception of life prolonging drugs that may extend patients’ survival by a few months but only if used in the early stages of disease. In contrast, recent stem cell based research suggests there are opportunities to repair and restore the injured nerve tissue by using stem cell therapy (i) as neurotrophic factors; (ii) cell transplants to replace lost cells or support those targeted by the disease process; (iii) recruitment of endogenous cells to replace those lost to the disease process; (iv) modification of the glial or inflammatory response; and/or (v) modification of the extracellular matrix and/or glial and immune reactions to minimize environmental barriers [3]. However, for neuronal disorder stages, when a critical number of neurons has been lost, the neuronal cell replacement for functional improvement of diseased tissue areas seems to be the only treatment option.

When searching for an ideal stem cell source, neural stem cells (NSCs) offer the most promising possibilities, as they can differentiate into various cell types of the nervous system. However, NSCs are situated in deep regions of the brain, and are usually isolated from aborted fetuses [4]. Therefore, they are not allowed for clinical applications due to enormous ethical concerns. In contrast, induced pluripotent stem cells (iPSs) as an autologous source have often been shown to be differentiable into functional neuronal cells and pre-clinical animal models speak for their feasibility [5]. But this stem cell source is still not clinically approved due to its potential for teratoma formation [6]. In contrast, adult mesenchymal stem cells (MSCs) are approved for clinical applications but to date their ability to differentiate *in vitro* into functional neuron-like cells was rarely reported [7–10].

Although it could be often shown that MSCs express neuronal markers like Nestin and ß-tubulin, and that their further neuronal induction led to enhanced neuronal phenotypic characteristics, it remains questionable if the plasticity of MSCs is sufficient for reliably generating functional neuronal cells [11, 12]. One of the main uncertainties results from the heterogeneous and inconsistent composition of isolated stromal cells. In order to up-concentrate the most potent stem cells out of heterogenous MSC populations, cultivation techniques were applied inducing more potent cells to accumulate in spheroids over the adhering cell layer. This phenomenon is commonly called as neurosphere formation and originally only referred to subventricular zone (SVZ) neural stem cells spontaneously forming spheroids upon *in vitro* addition of FGF and EGF in a serum reduced culturing process. These SVZ neural stem cells have the potential to differentiate into neurons, glia and astrocyte cells [13]. Although not sufficiently confirmed that MSC can be differentiated into each cell type: functional neurons, glia and astrocyte cells, the term “neuro-sphere” was adopted for adult MSC spheroid formation [14]. However, previous studies confirmed that MSC spheroid formation leads to up-concentration of highly potent stem cells. Recently, Mukai et al. demonstrated improved neuronal differentiation and migratory properties from umbilical cord (UC) derived MSCs when using SCS from this population [15].

According to our previous study, we demonstrate that a small portion of UC-MSCs behave other than typical MSCs, as these cells show no plastic adherence but spontaneously form spheroids and show pluripotent characteristics [16]. With this study we go one step further and demonstrate that UC-MSCs derived SCSs, are comprised of highly potent cells that gain neuronal functionality after simultaneous neuro-mechanotransductive and neuro-biochemical stimulation. Following the strategy that differentiation works most effectively when stem cells receive molecular and physical signals of the target tissue environment, we cultured the SCSs in neuronal inductive media on hydrogels that mimicked brain tissue. Gel stiffness was 0.5 to 1 kPa and provided a micro/nano-grooved surface to simulate structured brain tissue [17]. To investigate structure vs. stiffness as a mechanical cue, unstructured gels were also tested.

Whereas the brain specific stiffness is easily measurable and imitable, a specific topography is difficult to transcribe, as the direct environment of neurons is highly variable and dependent on their location. However, in accordance with previous studies, we simulated a tissue environment that should mimic the long thin shape of neurites from closely packed neighboring cells, as well as the often parallel alignment of axons and dendrites [18, 19]. Therefore, we designed a structure with alternating micro and nano-scaled grooves and imprinted this topography into the hydrogel gelatin meta-acrylate (GelMA).

The outgrowth and adhesion of SCSs on GelMA, in combination with biochemical induction induced neuronal marker expression and electrical activity, suggests that SCSs were neuronally differentiated. Remarkable was that *in vitro* neuronally differentiated SCSs strongly expressed *RBFOX3.* RBFOX3, an extensively described splicing regulator that is detectable in the developing mammalian brain cortex is known to be important to promote neuronal maturation, particularly through axon initial segment assembly and initiation of cell type specialization [20, 21]. In addition, we demonstrate that differentiated SCSs display neuron like functional properties. Namely, calcium imaging of SCSs constricted to the synapsin-expressing cells revealed a neuron like activity in around 65% of investigated cultures. SCSs exhibited a significantly higher density of active cells than adherent cells, allowing randomized patch-clamp investigations. Moreover, we confirm the electrogenic properties and excitability of differentiated SCSs, which were comparable to those of neurons.

## Materials and Methods

### Cell Isolation from UC

UC were received from University Hospital RWTH Aachen after obtaining the informed consent of the donor’s parents. Once in the laboratory, the UC was cleaned under the running water until residual blood was removed. Under sterile conditions, the UC was disinfected in 70% ethanol for 30 s and rinsed three times with Dulbecco’s phosphate-buffered saline (PBS), supplemented with 500 U/mL penicillin (PEN) and 500 µg/mL streptomycin (STREP). The UC was cut into small pieces with a length of about 1–2 cm. To avoid contamination of the subsequent cell culture with blood cells, areas that contained large blood clots were discarded and arteries and vein were carefully pulled out. For cell outgrowth the UC pieces were stuck to the petri dishes with the Wharton’s Jelly side down and covered with DMEM, supplemented with 15% fetal bovine serum (FCS), 100 U/mL PEN, 100 µg/mL STREP, 2.5 µg/mL Amphotericin B (Amp B) and 0.1% Ciprofloxacin hydrochloride (CFA). After 10–15 days of incubation at 37 °C in 5% CO2, the first cells migrated out of the tissue pieces. The first medium change took place after 5 days and then was carried out every 2–3 days. The tissue pieces were discarded after the outgrowing cells had reached 50% confluence. When UC-MSC reached confluence of approximately 80%, the cell culture was split using Trypsin/EDTA and spontaneously formed spheroids with a diameter round about 200 µm were taken out for further experiments.

### Ectodermal Trans-Differentiation

The ectodermal differentiation potential of WJ-derived cells was investigated by using the ‘StemMACS™ Trilineage Differentiation Kit, human’ from Miltenyi Biotec (Bergisch Gladbach, Germany). Spheroids were seeded in a collagen I-coated cell culture flask and cultivated in culture medium (CM) DMEM supplemented with 10% FCS, 100 U/mL PEN, 100 μg/mL STREP and 0.1% CFA at 37 °C and 5% CO2. Subsequently, cells were detached by trypsination after reaching 90% confluence and seeded at a concentration of 48,543 cells/cm^2^ onto ‘Corning® Matrigel® hESC-Qualified Matrix’-coated glass coverslips and treated with”StemMACS TM ecto-diff Medium” according to the manufacturer’s recommendation. The medium was changed daily and the cell morphology was monitored by phase contrast microscopy (Axiovert 40 CFL, Carl Zeiss Microscopy, Munich, Germany). On day seven the differentiation was assessed immunocytochemically by fluorescence microscopy (Biorevo BZ-9000, Keyence, Neu-Isenburg, Germany), regarding the expression of the ectodermal progenitor marker proteins Sox2 and Pax6. For the detection of nuclei, cells were stained with DAPI solution for 10 min.

### Manufacturing of Micro- and Nano-Structured GelMA Hydrogels

To generate biomimetic substrates with neuro-inductive topography, a polymeric template with the specific micro/nanostructure was produced using Roll-to-Roll UV-Nanoimprintlithography. A flexible Polyethylenterephthalat (PET) substrate was covered with an acrylate-based UV- curable resist. The resist was then structured by an embossing drum produced in ultra-precision diamond turning. Finally, the structured resist was cured by UV radiation, released, and collected by the rewinder. The manufactured structured film was then used to mold GelMA-hydrogels. GelMA-hydrogels were prepared in a water bath at 80 °C by dissolving 0.5% (w/v) 2-Hydroxy-4’-(2-hydroxyethoxy)-2- methylpropiophenone (Irgacure 2959) in PBS-/- for 5 min. The Irgacure 2959 solution was then used to dissolve 10% (m/v) GelMA at 80 °C for 5 min or until the mixture was completely dissolved. For this purpose, it was thoroughly mixed multiple times. The completed GelMA solution was pipetted onto the structured film or unstructured film (control sample) and cured in a LED Cube 100 IC UV irradiation chamber with a UV-wavelength of 365 nm (15mW/cm^2^) for 240 s at 21 °C. For drying, the hydrogel was placed in a desiccator filled with silica gel beads and dried for at least 3 days. Before the GelMA samples were used as substrates for neuronal induction, they were swollen in CM overnight and imaged with phase contrast microscopy (Fig. 3E).

### Seeding and Cultivation of WJ-derived SCS or aMSC on GelMA Hydrogels

The SCS or aMSC were applied on the GelMA hydrogels, either by transferring 500 µl of SCS suspension or seeding of aMSC in a concentration of 10,000 cells/cm^2^. To support the initial adhesion of outgrown cells or seeded cells, cell laden hydrogels were cultured in FCS containing CM for 48 h. Subsequently, the CM was aspirated and replaced with neural inductive media I (NIM I) containing Dulbecco’s Modified Eagle Medium/Nutrient Mixture F-12 (DMEM/F12), supplemented with 500 U/mL PEN, 500 μg/mL STREP, 2.5 μg/mL AmpB, 0.1% CFA, 1% Bottenstein’s N-2 formulation supplements (N-2), 2% Brewer’s B27 formulation supplements (B27), 50 μM 2-Mercaptoethanol, 20 ng/mL bFGF, 20 ng/mL insulin-like growth factor-1 (IGF1) and 20 ng/mL EGF. NIM I was changed every 2–3 days. After 14 days, NIM I was aspirated and replaced by ‘StemMACS Trilineage EctoDiff Medium’ (Miltenyi Biotec, Bergisch Gladbach, Germany), supplemented with 500 U/mL PEN, 500 μg/mL STREP and 0.1% CFA, for 7 days. Finally, cells were cultivated with Neural inductive media 2 (NIM II) to initiate neuronal maturation. NIM II consists of Minimum Essential Medium, alpha modification (α-MEM) and DMEM/F12 in a ratio of 1:1, supplemented with 500 U/mL PEN, 500 μg/mL STREP and 0.1% CFA, 2 mM L-Glutamine, 2% B27, 1% N-2, 1 mM cyclic adenosine monophosphate (cAMP) and 30 ng/mL neurotrophin-3 (NT3). Medium changes followed each third day, and the cultivation duration was between 14 to 16 days.

### RNA Isolation from SCS or aMSC

After the neural induction process, cells were rinsed twice with PBS-/- and 0.4 mL precooled TRIzol™ Reagent were directly added to the cell layer. The cells were detached form the GelMA hydrogel with a cell scraper and the cell lysate was collected with a P1000 pipette tip and transferred to a 1.5 mL reaction tube.

For complete dissociation of nucleoprotein complexes, 80 μL chloroform were added to the lysate and was incubated for 5 min. The aqueous phase, containing the RNA, was transferred into a new 1.5 mL reaction tube. Subsequently, 200 μL isopropanol were added to the aqueous phase, incubated for 10 min, and centrifuged for 10 min at 12,000 g and 4 °C. The RNA precipitate formed a pellet at the bottom of the tube, the supernatant was discarded, and the pellet was washed in 400 μL ethanol (75%). The sample was gently mixed and centrifuged for 5 min at 7,500 g and 4 °C. The supernatant was aspirated, and the RNA pellet was air dried for 10 min and resuspended in 20 μL RNase-free water. The RNA quality and yield were determined by ultraviolet–visible spectroscopy (UV–VIS) with the Implen NanoPhotometer® P330 (Implen, Munich, Germany) using a lid factor of 10 and a sample volume of 1 µL. The RNA was either processed immediately in downstream applications or stored at -80 °C.

### cDNA Synthesis

cDNA synthesis from 500 ng RNA per sample was performed using iScript™ Select cDNA Synthesis Kit (Bio-Rad Laboratories GmbH, Feldkirchen, Germany) according to the manufacturer´s instructions. The samples were gently mixed and incubated for 90 min at 42 °C, followed by a 5-min incubation step at 85 °C to heat inactivate the reverse transcriptase. cDNA products were processed immediately in downstream applications or stored at -20 °C.

### Real Time PCR

The quantitative real-time (RT) PCR assay was performed using the SsoAdvanced Universal SYBR® Green Supermix and the PrimePCR SYBR® Green Assay (Bio-Rad Laboratories GmbH, Feldkirchen, Germany) according to the manufacturer´s instructions. Each reaction mixture was prepared in triplet for each sample. PCR was performed in CFX 96 Touch™ Real-Time PCR Detection System (Bio-Rad, Feldkirchen, Germany). The applied thermal cycling protocol consists of an initial 2 min activation step at 95 °C followed by denaturation (95 °C for 5 s) and annealing (60 °C for 30 s) steps, repeated 40 times. The following primers were utilized to analyze the gene expression of the regulated genes *NANOG, POUF5, SOX2, RBFOX, NEF, CACNA1C, SNC9A, KCNB1*, and *NES* with assay IDs qHsaCED0046076, qHsaCED0046172, qHsaCID0036608, qHsaCID0014289, qHsaCED0042764, qHsaCED0045498, qHsaCID000960, qHsaCID0008219 and qHsaCED0001303. X-fold expression change of control cells in comparison to sample cells was investigated. Sample analysis was performed using the software ‘Bio-Rad CFX Maestro 1.1, Version 4.1.2433.1219’ (Bio-Rad Laboratories, Inc., Hercules, California, USA), which refers to Pfaffl, 2001 [57]. The house keeping genes (HKG) Glyceraldehyde 3-phosphate de-hydrogenase (*GAPDH*), Large ribosomal protein (*RPLO*), 18S rRNA and Beta-2-Microglobulin (*B2M*) with the assay IDs qHsaCID0015347, Hs99999902_m1, Hs99999901_s1 and qHsaCED0038674 were used to normalize gene expression of *NANOG*, *POUF5, SOX2*, or *RBFOX, NEFH, CaCNA1C, SNC9A, KCNB1, KCNQ5, NES*.

### Immunofluorescence Analysis

Immunofluorescence measurements were performed with Fluorescence Microscopy BZ-II Analyzer (Keyence, Frankfurt am Main, Germany). For immunofluorescence analysis of intracellular and nuclear markers Nestin, ß-III-tubulin, NeuN, and Nanog, Sox2, Oct4 and Pax6 'Tran-scription Factor Staining Buffer Set' by Miltenyi Biotec (Bergisch Gladbach, Germany) was used according to the manufacturer’s guidelines. For labelling of cell surface marker SSEA4, cKit, and CD49f cells were treated differently, samples were washed three times in PBS, fixed in 4% paraformaldehyde for 30 min at 4 °C and blocked with 10% normal goat serum and 2% BSA in PBS for 60 min. For primary antibody staining, cells were incubated with primary antibodies against SSEA4 (1:200, Invitrogen, Carlsbad USA), CD49f (1:100, Invitrogen), Nestin (1:200, Invitrogen) and ß-III-tubulin (1:500, Invitrogen), NeuN (1:500; Novus Biologicals, Centennial, USA), Nanog-PE (1:50, Miltenyi Biotec), Sox2-488 (1:100, Invitrogen), Oct4 (1:400, Invitrogen), Pax6-PE (1:11, Miltenyi Biotec), cKit-PE (1:50, Miltenyi Biotec) or against appropriate isotype control antibodies overnight, at 4 °C. The next day, cells were washed three times with PBS /0.1% BSA. Samples treated with antibodies without conjugated fluorochrome were treated with secondary antibodies Goat anti-Mouse IgG (H + L) DyLight 594 1:100 (Invitrogen, Carlsbad, USA) for ß-III-tubulin, Nestin, NeuN or Goat anti-Rabbit IgG (H + L) DyLight Goat IgG 1:500 (Invitrogen, Carlsbad, USA) for Oct-4 for 30 min at room temperature. Finally, the cell nuclei were stained for 10 min with 4´,6-diamidino-2-phenylindole (DAPI) diluted to 1 µg/ml in PBS-/- in a moist chamber at room temperature. The DAPI solution was removed, the tissue sections were washed 4–5 times with deionized water.

### Calcium Imaging

Calcium imaging experiments were mediated by genetically encoded calcium indicator (GECI) GCaMP6f that was expressed under the Synapsin promotor. This GECI was introduced by transduction with adeno-associated virus containing pAAV.Syn.GCaMP6f. WPRE.SV40 (a gift from Douglas Kim & GENIE Project to Addgene, viral prep #100,837-AAV9) under a viral load of 4.35 × 10^5^ GC/cell [20].

Calcium measurements were performed from 35 DIV on an inverted microscope Axio Observer.Z1 (Zeiss, Germany), with a 10 × objective (NA = 0.45). Every calcium imaging video was complemented by a bright-field snapshot. Illumination was via a Colibri LED system (Zeiss, Germany), with excitation bandpass filter (460–500 nm), and emission bandpass filter (500–530 nm). Visualization and image acquisition was conducted in Zen 2012 software, blue edition (Zeiss, Germany). Calcium videos were sampled at 6.25 to 100 Hz, with exposure time ranging from 150 to 10 ms, respectively. The image was binned 4 × 4.

The videos were processed in a home-made video-processing software in Python. Briefly, regions of interest were automatically detected by maximum intensity projection defined borders, followed by manual curation in SamuROI GUI [69]. From defined ROI borders, mean intensity traces were extracted. To prevent artifacts that arise from bleaching, mean intensity traces were normalized by the sliding 5^th^ percentile in a 2000 frames long sliding window. To emphasize calcium peaks, traces were detrended and filtered by the Butterworth low-pass filter at 3 Hz cut-off. 18 mM KCl was added to stimulate spontaneous activity in differentiated cells.

### Patch Clamp Technique

Patch-clamp measurements in whole-cell configuration were performed on 35 DIV cells, in a bath solution of (in mM): NaCl 120, KCl 3, MgCl_2_ 1, CaCl_2_ 2, HEPES 10. Patch pipettes were pulled out of 1.5 mm diameter borosilicate glass capillaries and filled with internal solution composed of (in mM): NaCl 2, KCl 120, MgCl_2_ 4, HEPES 10, EGTA 0.2, MgATP 0.2028. Both bath and internal solution had a pH adjusted to 7.3, but via NaOH and KOH, respectively. Experiments were performed without the liquid junction potential correction. Acquisition was done with respect to the reference pellet Ag/AgCl electrode. Cells were visualized under an AxioScope (Carl Zeiss AG, Germany) light microscope.

The signal was clamped, detected and conditioned by a HEKA EPC 10 double USB amplifier (Heka Elektronik GmbH, Germany), and the experiments were controlled by PatchMaster software (Heka Elektronik GmbH, Germany). Acquisition was performed at 10 kHz, and the input signal was hardware-filtered twice with Bessel filter: at 10 kHz and 3 kHz. Every cell undertook voltage-clamp and current-clamp measurements; in both, the resting membrane potential was kept at -60 mV and series of incrementing voltage or current pulses were applied. To construct IV curves, 520 ms long voltage pulses were applied in 20 mV increments, starting from -120 mV. Current-clamp experiments included fifty 50 ms rectangular stimulation pulses, with amplitude starting from – 200 pA and successively increasing by 20 pA. Analysis was performed with a custom-made Python script.

## Results

### Spheroid Formation from UC-MSC Culture and Pluripotent Characteristics of SCSs

**Fig. 1:**
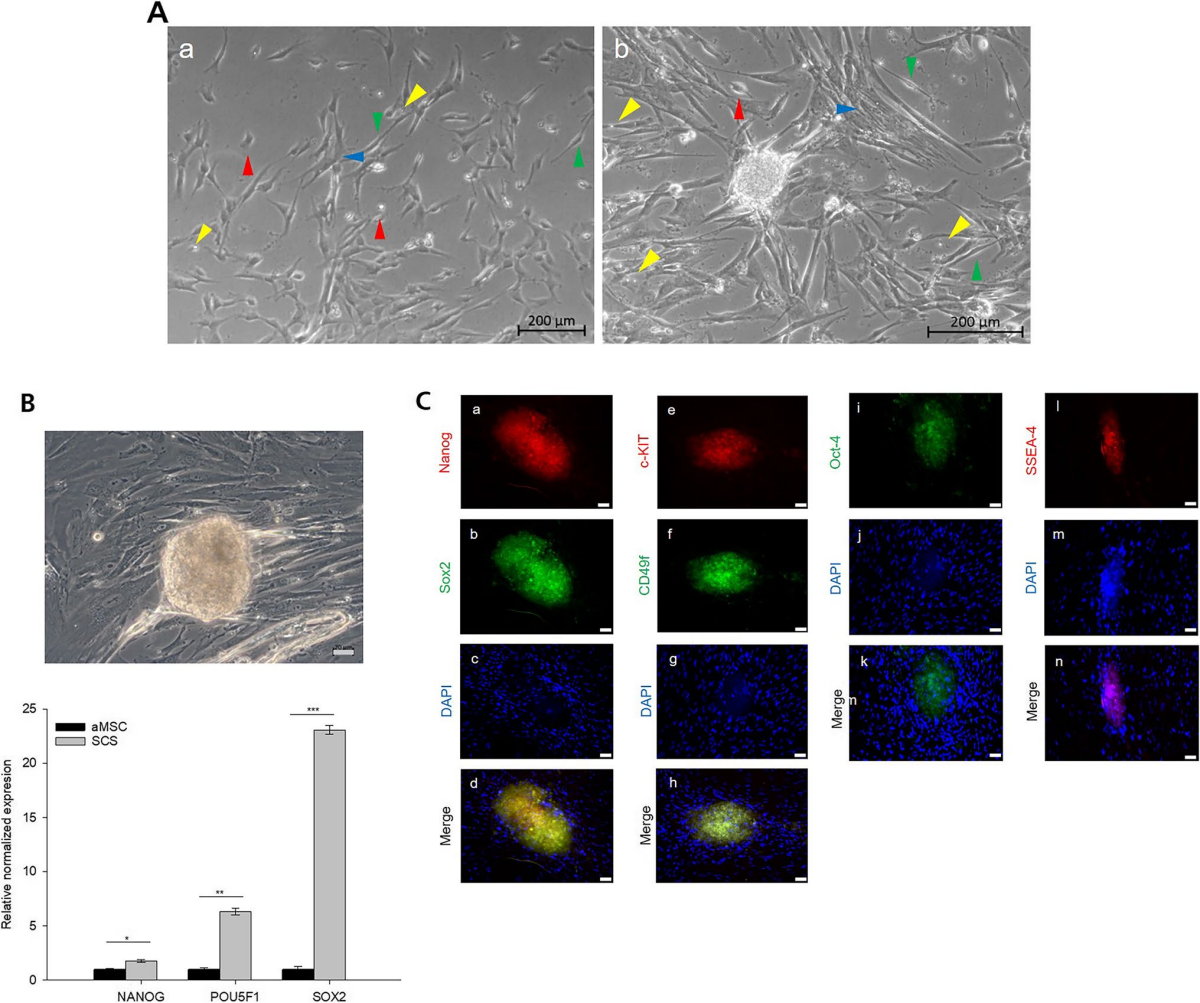
**Morphology, gene and protein expression of SCS versus aMSC derived from WJ tissue**. **A** Morphology of cells from the WJ region of the UC. Cells were obtained by outgrowth from WJ tissue pieces on TCP. The cell morphology was very heterogeneous, and the cells started to form colonies that developed into SCS as the cell density increased (Figure from 1A, a to 1A, b). Three major morphological cell shapes could be identified: In both cell populations, cells with an elongated spindle-shaped (green arrowheads) and a large flattened (blue arrowheads) morphology were observed, both characterized by a high cytosol-to-nucleus ratio. Furthermore, cells with a stellate to triangular shape (red arrowheads) and smaller roundish cells (yellow arrowheads) with a low cytosol-to-nucleus ratio were detected. The scale bars represent 200 μm. **B** Gene expression analysis of SCS originating from WJ-tissue. SCS were examined for their gene expression of NANOG, POU5F1 and SOX2. Gene expression data were normalized by means of the housekeeping genes GAPDH and B2M. (1B, bottom). Micrograph of a typical SCS collected for gene analysis, scale bar 20 µm. (1B, top) Gene expression data of aMSC (black bars) as control. The error bars indicate the standard deviation of three technical replicates. Significance in comparison to control was analyzed by one way ANOVA testing. * indicates *p* < 0.05, ** indicates *p* < 0.01 and *** indicates *p* < 0.001. **C** Protein expression analysis of Nanog, Sox2, c-KIT, CD49f, Oct-4 and SSEA-4 in SCSs and underlying aMSCs. The nuclei were stained with DAPI. Scale bars represent 50 µm

After outgrown from UC tissue pieces, the mixed stromal UC-WJ-derived cells were cultivated on tissue culture plates in FCS containing culture media (CM) until confluency was reached and spheroids were spontaneously built. According to the ISCT guidelines, the adherent cells fulfilled the minimal criteria for MSCs over all investigated passages from P0 to P5, and express all MSC phenotype related markers (supplemental data). In addition, the co-existence of hematopoietic stem cells was investigated by measuring CD14, CD20, CD34 and in fact CD45 and CD34 positive cells were always detectable in low numbers (S1). Figure 1A, a illustrates cells that were directly grown from UC-WJ tissue pieces. Most of the migrating cells display an elongated or large flattened morphology, but there exist also a minor portion of cells with triangular or stellate shape or small-round morphology with a low cytosol to nucleus ratio (Fig. 1A, b). After four weeks of cultivation, the small round cells collectively accumulated at the surface of adherent cells and organized into spheroids (one representative sight field from one of three donors tested are shown in 1A, b and 1B, top). These spheroids remained attached to the adherent cell layer (1B, top) or floated freely in the culture media supernatant.

In order to investigate the effect of SCS formation with respect to accumulation of cells with pluripotent characteristics, we compared the gene expression of *NANOG*, *SOX2* and *POU5F1* from SCS forming cells against adherent MSCs (aMSCs). Figure 1B demonstrates with one representative result out of three donors tested thatin comparison to aMSCs the gene expression of *NANOG*, *SOX-2* and *POUF-1* were significantly enhanced by 1.77- fold, 23.09-fold and 6.31-fold, respectively, in SCS. Further it could be analyzed that free floating spheroids stronger express pluripotency genes than adherent spheroids (supplemental data; S2). Immunostaining analysis confirmed the existence of pluripotent cells located in spheroids at the protein level. As demonstrated in Fig. 1C, only cells within spheroids were positive for Nanog, Sox-2, Oct-4, cKit, CD49f and SSEA-4. Surrounding the SCS, larger adherent cells were not labelled by the pluripotency associated marker antibodies. Isotype exhibited no fluorescence signals (supplemental data, S3). In addition, images with higher resolution show within spheroids single cells positive for Nanog, Sox-2 and Oct-4. (supplemental data, S4).

### Differentiation Towards Early Ectodermal Cells

**Fig. 2:**
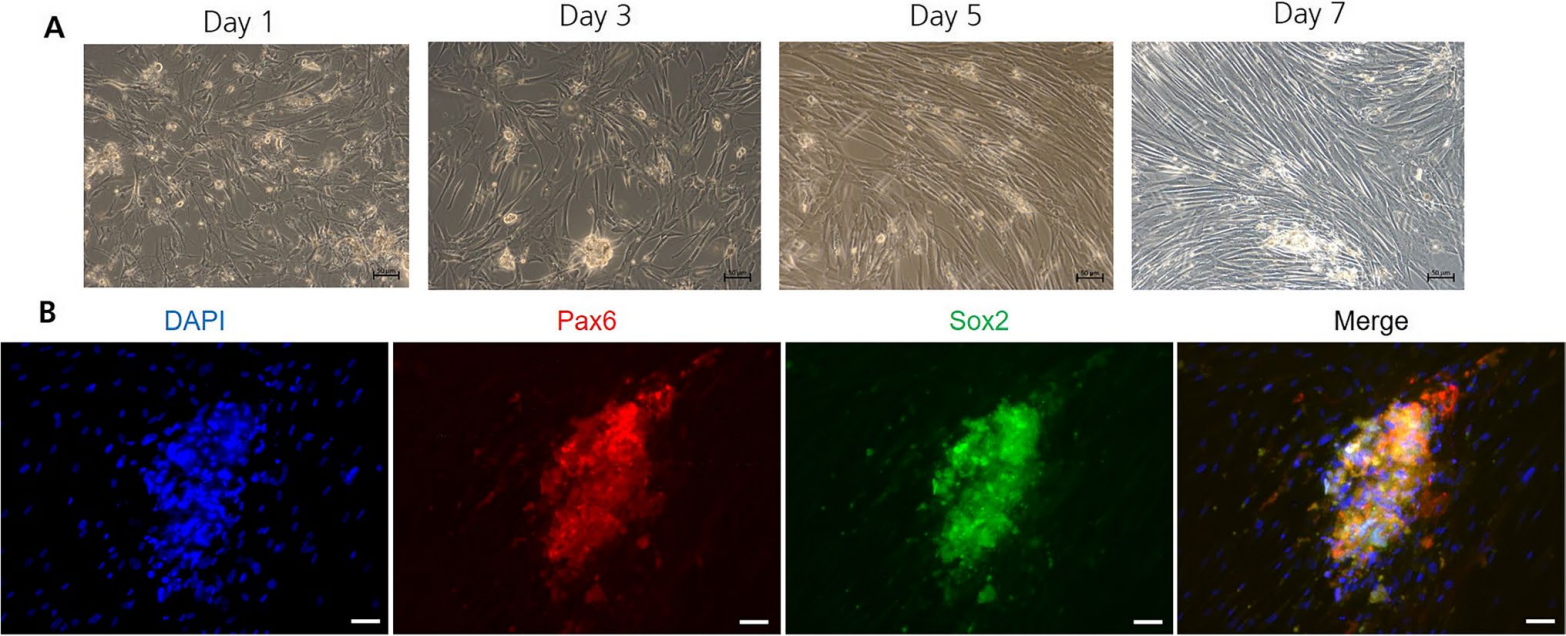
**Ectodermal differentiation of SCS and aMSC during the induction**. **A** Growth on Matrigel. Pre-selected UC MSC cells were seeded on Matrigel and cultivated in ectodermal induction medium for six days and the morphology was investigated for seven days. Scale bars represent 100 µm. **B** Early ectodermal lineage differentiation. Ectodermal lineage was confirmed by immunofluorescence for Pax-6 and Sox-2. The cell nuclei were counterstained with DAPI. The scale bars represent 50 µm

UC-MSC cells were analyzed with respect to their ability to undergo differentiation into ectodermal progenitor cells. According to the manufacturer’s recommendation, aMSC were seeded at a high cell density on Matrigel coated glass slides and treated with an inductive CM for six days. On day seven, the expression of early ectodermal markers was investigated by immunocytochemistry. Figure 2A demonstrates with one representative result out of three donors tested the morphological changes of the cells during the differentiation process. On the first day, the cells exhibited a mixed morphology. From day 1 to day 5, the morphology of the majority of cells became more and more elongated. However, a small amount of small and tiny cells showing a roundish morphology remained unchanged in their morphology. By day 7, the number of elongated cells increased overall, and the cells partially aligned in parallel. Simultaneously, the amount of smaller and tiny roundish cells accumulated and the cells built-up spheroids on the surface of the adhering cell layer. Immunostaining of Sox2 and Pax6, ectodermal lineage-specific transcription factors, revealed expression only in the spheroid forming cells and not in the surrounding adherent cells (Fig. 2B). The cell nuclei were counterstained with DAPI to indicate the presence of Pax6 and Sox2 negative aMSCs.

## Production of Scaffold

**Fig. 3:**
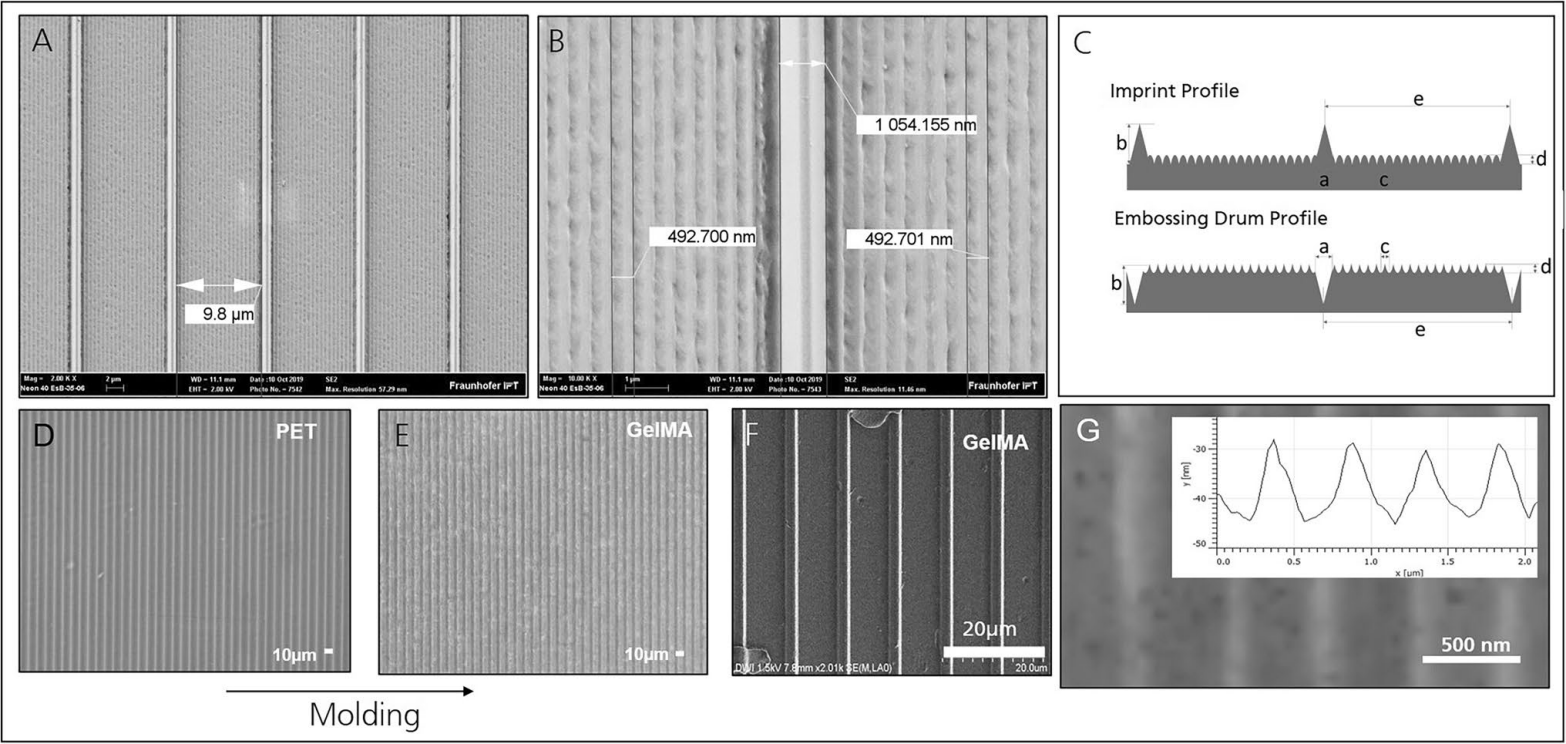
**Micro- and nano-grooved topography of the biomimetic template**. **A** Template for micro and nano grooves. SEM images of the PET template at low magnification, showing the pattern of micro and nano grooves. **B** Template single unit. High magnification SEM of the PET template shows individual nanogrooves with frayed edges in one of the pattern units. **C** Schematic representation of the drum and molded PET. The master structure with micro- and nano-grooves shows the structure with dimensions and parameters of the indices listed in Table 1. **D** Phase contrast microscopy of PET master mold. The scale bar represents 10 µm. **E** Molded hydrogel. Phase contrast microscopy image of micro- and nano-grooved GelMA molded from the PET template. The scale bar represents 10 µm. **F** Surface structure in microscale. FESEM image of GelMA impression from PET master mold. The scale bar represents 20 µm. **G** Surface structure in nanoscale. Atomic force measurement measured grooves in a period of 500 nm and a depth of approximately 15 nm. The scale bar represents 500 nm. Grayscale indicates detected heights

In the first step, an embossing drum was structured with the described micro- and nanotopography in an ultraprecision diamond-turning process in which both structure types were directly resulting from the machining kinematics. The drum was embossed by roll-to-roll UV-Nanoimprintlithography into a polymeric substrate, which served as a replication-template for the subsequent steps. For a comprehensive neuronal differentiation induction, gelatine-methacrylol GelMA substrates were molded from a template that was structured with a micro- and nano-grooved topography. Figure 3 shows images of the template surface imaged at 2000x (3A) and 10,000x (3B) magnification. A schematic structure of the polymeric template with micro- and nanogrooves is shown in Fig. 3(C). Each ridge between the micro-grooves contained further nanogrooves and the corresponding parameters are listed in Table 1. The micro and nanoscale topographies were measured using scanning electron microscopy (SEM). Figure 3E demonstrates GelMA molded from the structured PET template. The volumetric molecular dimensions of the GelMA are approximately 400 × 400x400 pm^3^. Thus, it is generally suitable to precisely replicate structures on the nanometer scale. Its potential to replicate the targeted features is validated by FESEM (Fig. 3F) and AFM (Fig. 3G) measurements of the molded GelMA. The replication of the nanogrooves is clearly visible and can be seen in Fig. 3G.

**Table 1:**
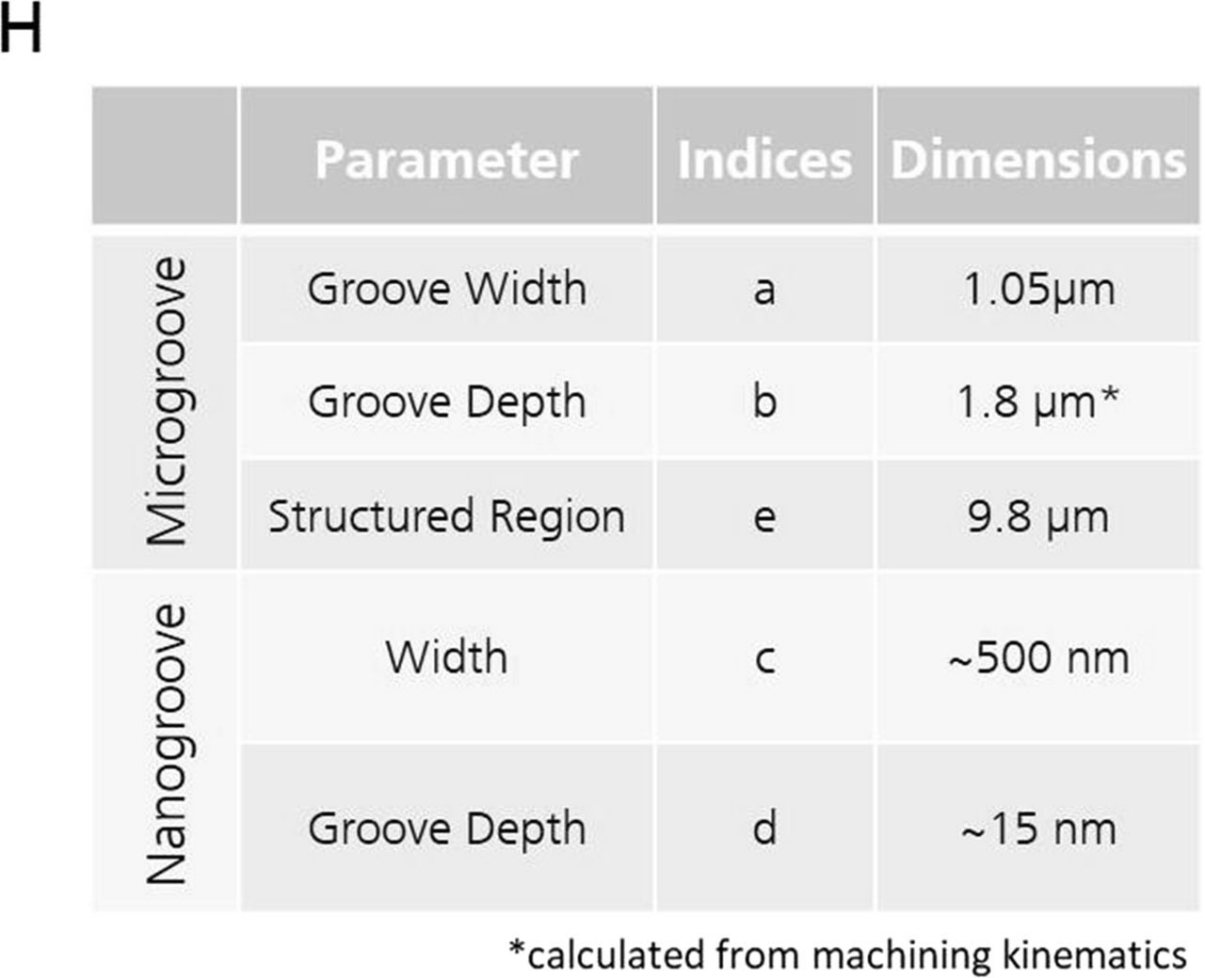
Dimensions of the GelMA structures. The height, width, and pitch of the structures achieved in GelMA were measured in by FESEM and AFM and are listed according to the indices provided in Fig. 3C. Calculated dimension from machine kinematics is indicated by the *

### Morphology of Neuronal Differentiated SCS in Comparison to aMSC

**Fig. 4:**
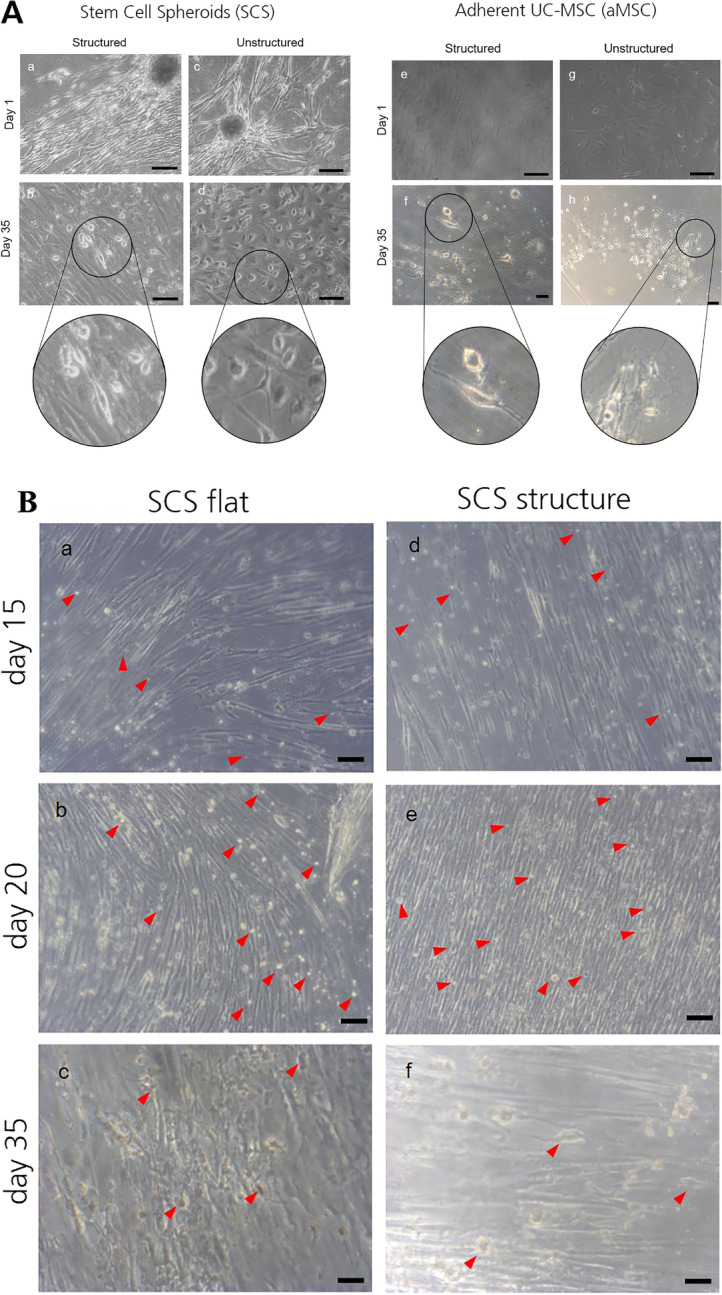

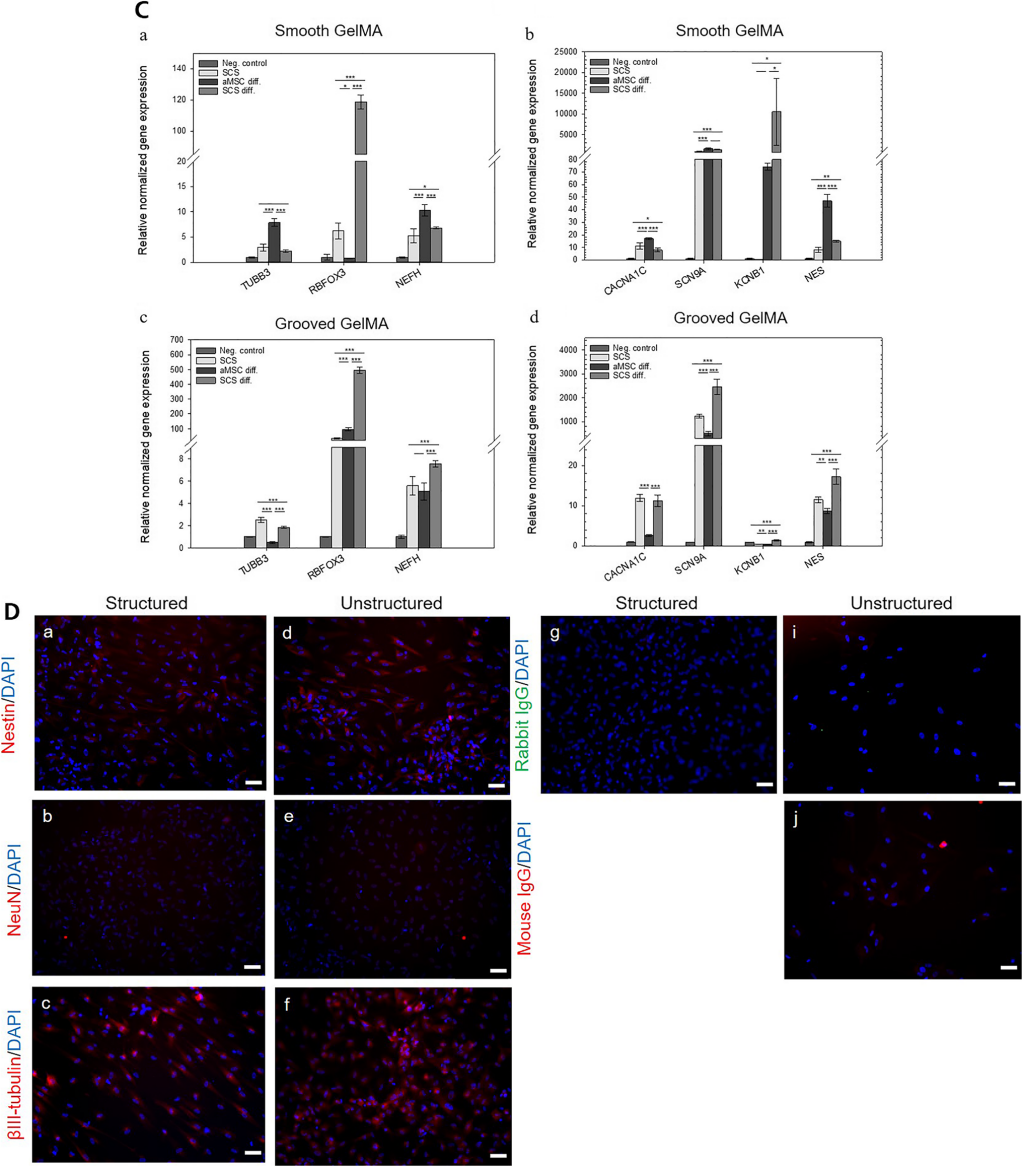
**Biomimetic and biochemical neuronal induction of SCS and aMSC**. **A** Differentiation on GelMA. Phase contrast images of SCS or aMSC during neuronal induction through biomimetic (structured GelMA, a, b, e, f) and biochemical cues (c, d, g, h), smooth GelMA provides only biochemical cues. Cues were provided for 35 days. Magnification shows cells with neuronal morphology. The scale bars in a and c represent 200 µm, in b, d, e, g, and h 100 µm, and 50 µm in f. **B** Morphological changes after three biochemical induction periods. Day 15 NIM I media was exchanged against early ecto-diff induction media. The red arrow-heads indicate tiny and small roundish cells in a & d. On day 20 ectodermal induction is finished, red arrowheads mark cells with roundish or slightly oval cell shape in b & e. On day 35, 15 days after the last induction step with NIM II, a few cells show neuronal morphology (red arrowheads in c & f). The scale bars represent 100 μm. **C** Neural gene expression of SCS or aMSC after biomimetic and biochemical neuronal induction. Relative normalized gene expression of NEFH, NES, RBFOX3, TUBB3, CACNA1C, KCNB1, KCNQ5 and SCN9A in Jurkat cells (as negative control) against undifferentiated SCS, differentiated aMSC and differentiated SCS cultured on smooth (a and b) or micro/nano grooved GelMA (c and d) hydrogels for 35 days. The gene expression data were normalized by means of the housekeeping genes GAPDH and B2M. The error bars indicate the standard deviation for three technical replicates. Significance in comparison to control was analyzed by one way anova testing. * indicates *p* < 0.05, ** indicates *p* < 0.01 and *** indicates *p* < 0.001. **D** Expression of neural marker proteins examined by immunofluorescence staining. SCS were seeded onto structured (a-c and g-h) and unstructured (d-f and i-j) GelMA hydrogels and cultured over a period of 35 days in neural induction media. Cells emerging from the spheroids were positively stained for nestin (a and d), NeuN (b and e) and β-III-tubulin (c and f). The nuclei were stained with DAPI and the isotype control stains (g, i) exhibited no fluorescence and h and j weak fluorescence signals. The scale bars represent 50 µm

After confirming the pluripotent character and ability to differentiate into an ectodermal direction, we investigated if SCSs are able to differentiate into active neuron like cells through biomimetic and growth factor induction. As control we seeded aMSC after plastic adherence in a density of 10,000 cells/cm^2^ and submitted them to the same biomimetic cues and growth factors. With one result representative out of three donors tested Fig. 4A, a and A, c, illustrate that cells grown out of the SCSs began to proliferate and migrate after the first days of culturing. During further cultivation SCSs aligned to the grooves and showed a longer and thinner cell shape than cells adhered to flat GelMA (4A, b vs. 4A, d).

After 14 days of NIM I induction SCSs showing elongated morphology and many roundish cells of tiny or small size were apparent (Fig. 4B, a and d). Subsequently stimulation with Ectodiff media enhanced proliferation of the elongated cells and more roundish cells of small size became apparent (Fig. 4B, b and e). With the last induction process from day 20 to 35 the cell density was reduced, and some cells showed neuronal morphology (Fig. 4B, c and f). aMSCs behaved differently. After cell seeding in a high density, most of the cells became detached from GelMA and went into apoptosis and necrosis after NIM I induction (data not shown). However, the remaining cells survived subsequent Ectodiff and NIM II induction and finally a small number of them showed neuronal morphology (Fig. 4A, f and h).

### Expression of Neuronal Marker Genes

The effect of the various combinations of cell source and biomimetic plus biochemical or biochemical induction factors alone was evaluated for two donors by gene expression profiling after 35 days of cultivation. The relative gene expression of *NEFH, NES, RBFOX3, TUBB3*, *CACNA1C, KCNB1* and *SCN9A* were determined for SCSs and aMSCs and were compared to the normalized gene expression from Jurkat cells. For normalization, housekeeping genes *GAPDH* and *B2M* were taken, and representative example of RT analysis is shown. When grown on smooth GelMA, differentiated aMSCs showed a higher expression of *TUBB3, NEF, CACNA1C*, *SCN9A* and NES than SCSs before or after differentiation, Fig. 4C, a & C, b (p < 0.001, ANOVA). In contrast, neuronal splicing regulator *RBFOX3* was expressed significantly more in differentiated SCSs than in any other cell type tested. Pre-differentiated SCSs showed elevated *RBFOX3* compared to aMSCs (6.23 vs. 0.79), but was dwarfed by a post-differentiation expression level in SCSs of 118.69. Unlike calcium channel *CACNA1C*, which had higher expression in aMSCs than either differentiated or undifferentiated SCSs (17.02 vs. 7.99 and 11.12, respectively), the sodium channel *SNC9A* was elevated in differentiated aMSC and differentiated SCS cells but remained low in undifferentiated SCSs (1593 and 1412 vs. 875, respectively). The average expression of *KCNB1* in differentiated SCSs was very high (10,515.21), the high variability of *KCNB1* expression among differentiated SCS samples rendered comparison to aMSC (74.27) insignificant and low significant with ( *p* < 0.05) in comparison to SCSs (0.46).

When cells were grown on structured GelMA in addition to chemical differentiation, not only the absolute levels of expression but the trends across cell type changed, Fig. 4C, c and C, d. Here, aMSCs never showed the highest expression levels. On grooved GelMA, differentiated SCSs had the highest expression of *RBFOX3, NEF, SNC9A*, *KCNB1*, and *NES* versus all other cell types. On smooth GelMA, *TUBB3* was not suppressed in aMCS, while on grooved GelMA SCS *TUBB3* levels were suppressed to a significant level during differentiation and levels in aMCSs were suppressed even more strongly. Grooved GelMA also reduced *NEF* expression in aMSCs below the levels in SCSs, despite increasing the significance of the rise in *NEF* expression during SCS differentiation (5.56 to 7.53 on grooved vs. 5.26 to 6.82 on flat). Though aMSC expression of *RBFOX3* became higher than undifferentiated SCSs (95.34 vs. 33.19) both were significantly less than differentiated SCSs at 495.88 on grooved GelMA, suggesting that grooves increase *RBFOX3*, in both cell types, but in SCS this is on top of the chemical effect. The level of *CACNA1C* was elevated in both differentiated (11.29) and undifferentiated SCSs (11.95) compared to differentiated aMSCs (2.60), this suggests grooves turn down *CACNA1* in aMSCs.

On both smooth and grooved GelMA, undifferentiated SCSs expressed more *TUBB3* than differentiated SCSs, though on smooth GelMA this difference was not significant (2.49 vs. 1.85, and 2.94 vs. 2.41, respectively). On grooved GelMA, the expression of *CACNA1C* did not change significantly during SCS differentiation, in contrast to the reduced *CACNA1C* during SCS differentiation on smooth GelMA (4C, b, *p* < 0.05). The increase in *NEF*, *NES*, and *KCNB1* during SCS differentiation became more significant when SCSs were differentiated on grooved GelMA instead of smooth GelMA (*p* < 0.05 vs. *p* < 0.001, p < 0.01 vs. *p* < 0.001, and *p* < 0.05 vs.* p* < 0.001, respectively).

In addition, with the same samples the relative gene expression of TMEM119, DCX and NEUROD1 were analyzed to demonstrate that the induction protocol renders glial cell differentiation. As a result, NIM1/NIM2 induction of SCS cultured on smooth GelMA reveal 9.45xfold expression of TMEM119, 8.94 × fold expression for DCX and 8.91 × fold expression for NEUROD1. In comparison to smooth GelMA, SCS cultivation on grooved substrates gained higher expression rates of specifically 12.71, 17.83 and 10.29 for respectively TMEM 119, DCX and NEUROD1 (supplemental data, S6).

### Protein Expression of Relevant Marker Confirming Neuronal Differentiation

To confirm gene expression data on a protein level, we attempted immunofluorescence on SCSs and aMSCs after 34 days of neuronal biomimetic and biochemical induction. aMSC immunostaining was impossible due to the low number of cells which were left on GelMA after 35 days. The few remaining cells (as in Fig. 4A, f & h) detached completely during handling for the staining procedure. In contrast, SCS cells adhered more stably to GelMA during the induction process and the immunostaining procedure did not lead to loss of sample from the hydrogels. The post differentiation SCS cells were examined for the expression of the neural-associated markers Nestin, NeuN, and ß-III-tubulin. The results in Fig. 4D, illustrates with one representative example out three donors the cells on structured (a-c, g-h) and unstructured (d-f, i-j) hydrogels. Both cell populations expressed all measured markers. The cell nuclei were stained with DAPI and showed a blue signal. Isotype control stains, Rabbit IgG (g and i) did show no positive fluorescence signals but Mouse IgG (h and j) show slightly positive fluorescence signals.

### Calcium Imaging

**Fig. 5:**
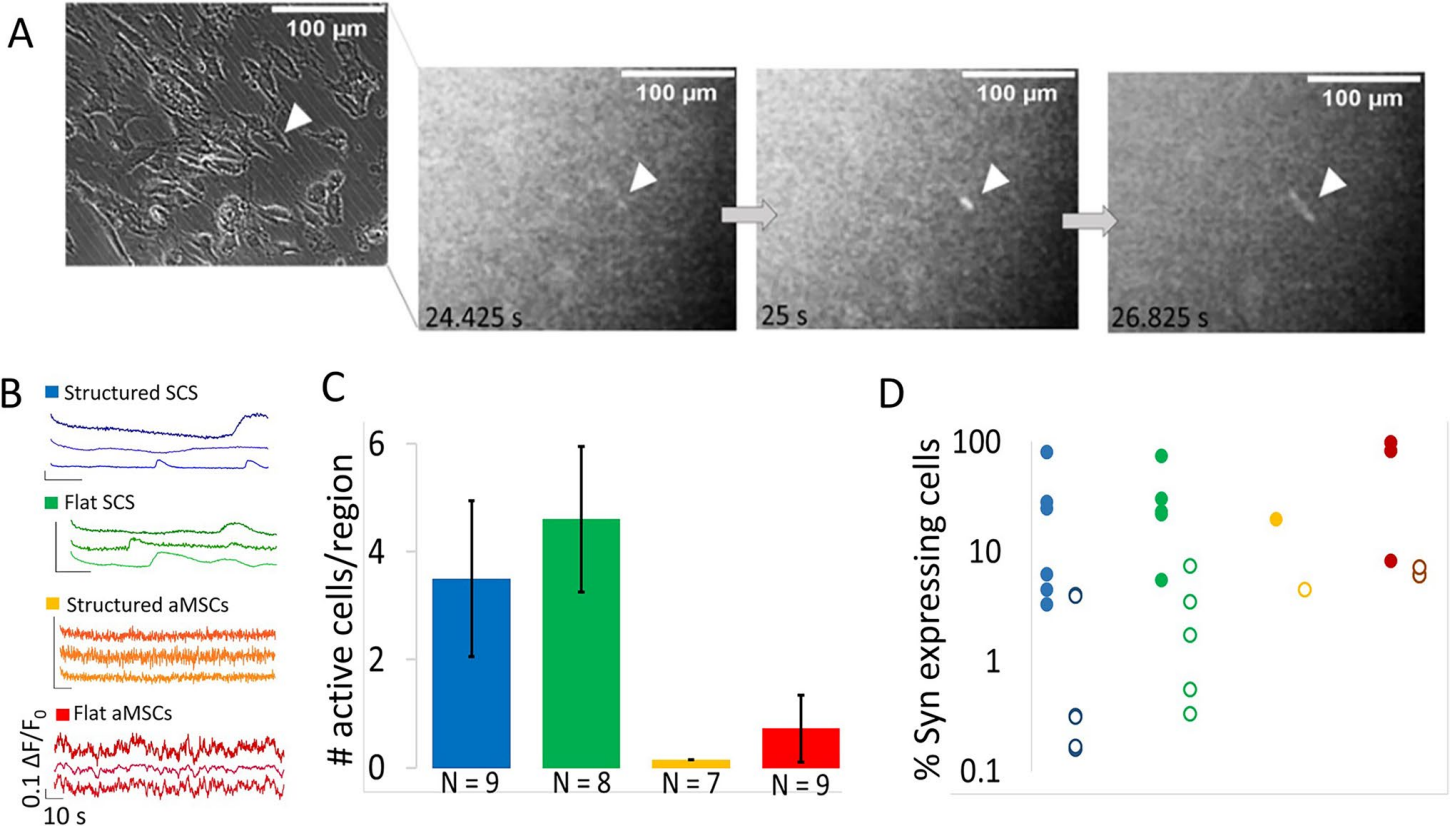
**Calcium analysis of differentiated SCS and aMSC**. **A** Calcium imaging. Left – brightfield image of cells. Right—three frames of GCaMP6f fluorescence response to a calcium event triggered by addition of 18 mM KCl. White arrow marks the active cell in all micrographs. **B** Exemplary filtered calcium traces from multiple cultures. Blue—SCSs on structured gels, green—SCSs on flat gels, orange—aMSCs on structured gels, red—aMSCs on flat gels. Color code remains the same throughout. **C** Distribution of active cells per imaged region. Number of active SCS and aMSC on structured and flat gels per 2.34 mm^2^. **D** Calcium event rates. Fraction of SCS and aMSC synapsin expressing cells with calcium events (filled dots) and fraction of cells with calcium events out of total cells identified in brightfield images (empty circles). Due to the log scale, cultures with no active cells are not plotted

Calcium imaging was used to screen hundreds of differentiated cells on structured or unstructured GelMA. Since the calcium activity was monitored using the genetically encoded calcium sensor GCaMP6f, under the control of the human synapsin promoter [22], we favor detecting calcium events from cells in a neuronal fate over those that have acquired muscle-like characteristics. Calcium events could be triggered by the addition of KCl to depolarize the cell membrane, suggesting voltage responsiveness of the calcium events, similar to action potential responses generated in neurons by KCl depolarization. Figure 5A shows frames from a calcium imaging movie indicating the appearance and disappearance of calcium signal in a SCS cell that has taken an elongated shape.

Estimation of spontaneous activity was performed over multiple random regions per culture. Addition of KCl limited the screening to individual regions in a short time interval, since its prolonged treatment reduces the action potential-associated calcium events. Investigated cells were considered as active if they showed at least one spontaneous or KCl-induced calcium spike over the course of 1.5 min of recording. Figure 5B shows that calcium events with varying dynamics were visible in SCSs and aMSCs. Among them, SCSs grown on structured GelMA showed the strongest calcium signals. Interestingly, the kinetics of calcium events differed between SCSs and aMSCs. SCSs grown on structured and flat GelMA displayed broader calcium signals, several times wider than those of aMSCs. Both slow and fast calcium signals are found in native cultures of human neurons [23, 24]. However, slow and broad transients, typical for SCSs, grown on both flat and structured GelMA, are more comparable to those of neurons derived from human iPSCs at similar culture ages [25].

For SCSs grown on structured GelMA, calcium signals were detected in 67% of investigated cultures (*N* = 9), while around 62% of SCS cultures on flat GelMA were active (*N* = 8). Among the active cultures, SCSs on structured and flat gels had 3.5 and 4.6 active cells per imaged region, respectively. In contrast, recordings from aMSCs on GelMA most often showed no cells with active calcium signals. On average, less than one active aMSC cell could be detected per region investigated (Fig. 5C). When a GCaMP6f expressing cell was detected in an aMSC culture, it was likely a single cell, representing less than 5% of the total cells identified in brightfield imaging. In cultures of SCS and aMSCs, if a cell expressed GCaMP6f under the hSyn promoter, it was likely to show active calcium signals in response to KCl stimulation (Fig. 5D, filled circles). However, more cultures of SCSs showed higher fractions of active cells (Fig. 5D, note cultures with zero cells showing calcium events do not register on the semi-log scale).

### Functional Estimation of Neuron-Like Properties by Patch-Clamp Measurements

**Fig. 6:**
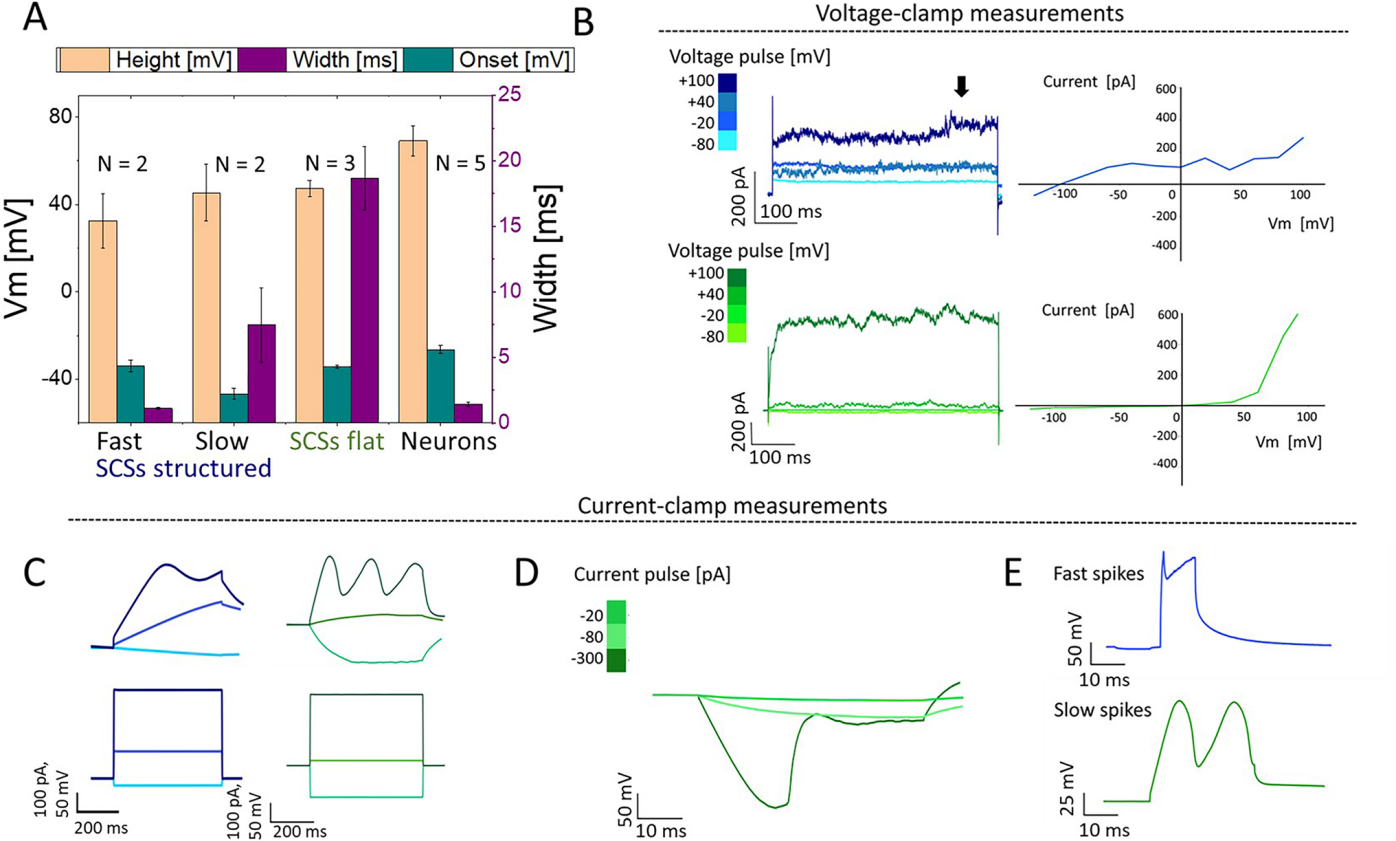
**Patch Clamp analysis of differentiated SCS**. **A** Quantification of active electrical properties. Comparison of spike amplitudes, onsets and widths for SCSs on structured and flat gels vs. cortical neurons. Spikes of SCSs grown on structured gels were further separated into fast and slow based on their durations. **B** Current responses. Current response of differentiated SCS when rectangular voltage pulses were applied by patch pipette in heat-map defined voltage steps (left). Black arrow indicates the time point used to generate IV curves (shown on the right). **C** Active cell responses. Examples of the voltage responses (top) from cells on structured (left) and flat (right) gels during patch clamp controlled current pulses (bottom). Threshold properties can be seen in cells on both types of substrates. **D** Voltage non-linearity induced by strong hyperpolarization. At -300 pA pulse, there is an HCN channel-like dip in voltage response, absent from responses induced by weaker stimuli. **E** Different induced spiking events. Whereas fast spikes (top) were detected only in SCSs grown on structured gels, injection of + 500 pA induces slow spikes in SCSs grown on flat and structured GelMA (bottom)

Furthermore, patch-clamp measurements in whole-cell configuration were performed to estimate electrogenic properties of SCSs. Of the 19 regions of aMSCs examined by calcium imaging, only 4 regions had any cells with calcium activity and two of those fields had only a single cell. It was therefore not feasible to randomly patch-clamp aMSCs to further characterize their activity. Figure 6 contains measured voltage and current responses of differentiated SCS cells upon current and voltage stimulation, respectively. In current-clamp, we looked for active, all-or-none voltage events evoked by injection of depolarizing currents [26–28]. In voltage-clamp, we looked for rectifying currents that are a hallmark of voltage-sensitive ion channels [27]. Voltage rectification can be easily spotted as deviation from a straight line in the I-V curve (Fig. 6B) [27].

Out of 10 patched SCSs grown on structured gels, 4 cells showed at least one electrogenic property in current-clamp or voltage-clamp mode (4 cultures), while for SCSs grown on flat gels, these properties were detected in 3 out of 6 cells (2 cultures). As shown in Fig. 6B, voltage-clamp measurements confirmed the voltage rectification and the presence of voltage-gated channels. During the injection of depolarizing voltage pulses, we detected a fast-activating, inactivating inward current followed by a slow-activating, non-inactivating outward current (Fig. 6B, left). For interplay and kinetics, these responses correspond to action potential (AP) carrying, sodium and potassium currents, respectively [27, 28].

Current-clamp measurements verified the presence of excitability and all-or-none behavior, as there were voltage responses evoked by depolarizing current in a threshold-dependent manner (Fig. 6C). The evoked responses displayed regeneration, since the membrane potential returned to the initial value after the beginning of a stimulation pulse. The evoked spikes showed similar onset potential to cortical neurons, however, their peak-to-peak amplitude ranged from 30 to 60 mV, which is smaller than 80 mV, calculated for average cortical neurons’ action potentials (Fig. 6A). Based on the kinetics of evoked spikes, we could distinguish two types: fast and slow events (Fig. 6E). With half-height width of 1.1 ms, fast events were comparable to APs. On the other hand, slow spikes were 10 times wider. While SCSs on structured gels displayed both fast and slow spikes, only slow spikes were detected in SCSs grown on flat gels. Furthermore, one multi-spiker cell was identified among SCSs on flat gels (Fig. 6C). This does not rule-out the presence of multi-spiker cells in SCSs on structured gels, but suggests differentiation into a multi-spiker cell is a rare event. Moreover, we detected another type of non-linearity in current-clamp measurements from flat and structured gels (Fig. 6D). The recorded potential displayed a dip towards hyperpolarizing values only at strong hyperpolarizing stimuli. Membrane potential recovered after the end of hyperpolarizing current injection. The kinetics and appearance of this hyperpolarization strongly resemble the hyperpolarization-activated inward current mediated through HCN channels, well-characterized for brain-stem [29] and cortical neurons [30].

At this point, we can only speculate about the presence of multi-spikers on structured gels, due to the low sample size and overall blindness to the calcium signal. However, in a proof-of-concept fashion, we demonstrate that SCSs can differentiate to excitable and electrogenic cells, with responses that are akin to those reported for cortical neurons [26, 28].

## Discussion

Neuronal replacement therapies as treatment for central and peripheral nerve disorders are still an unachieved goal. Previous studies reported differentiation of UC-MSCs into neuronal cells by demonstrating neuronal marker expression, secretion of neuronal factors, or both. Yet these claims weren't supported by examination of neuron-like functionality *in vitro* [5]. However, a lot of animal experiments demonstrated that application of UC-MSCs [31] or neuronal pre-differentiated UC-MSCs, which were in some cases genetically modified, induced beneficial activities at neuro-degenerated regions [32–34]. While not yet elucidated if UC-MSC are capable to differentiate themselves into functional neurons *in vivo*, it is well accepted that they express paracrine neurotrophic factors and cytokines that locally induce neurogenesis of remaining neurons, stop the course of neurodegenerative disease and heal damaged regions through anti-apoptotic, immunomodulatory, and anti-inflammatory effects [7, 31, 35].

With this study we wanted to further investigate the yet unresolved mystery if adult MSC from UC tissue have the potency to differentiate into active neuron like cells. The most common view is that cells of mesodermal tissues such as the UC can acquire properties of ectodermal cells by transdifferentiation and thereby become differentiable into neuronal cells [36]. Here we focus rather on the pluripotent stem cell fraction of the UC which were already discovered in our last study [16]. This pluripotent stem cell fraction consists of tiny roundish stem cells that were seen as single cells directly attached to large adherent stromal cells that were attached to the plastic. The fraction was enriched in spontaneously formed SCS after long term cultivation. Thereby, an intensive characterization of these tiny cells revealed that they express markers comparable to very small embryonic like stem cells (VSELs) found in UC blood [37, 38], such as CXCR4, CD133, SSEA-4, SSEA-1, OCT-4, Nanog, and SOX-2. Previous research studies suggested that VSELs, similar to PGCs, originate from epiblast during embryonic development, [39–41] and that VSELs from the embryonic period of life persist in human adult tissues and organs in a quiescent state [42]. Their molecular status has already been well characterized in both humans and mice. Evidence shows that VSELs are related to the germinal lineage and express several markers common to PGCs, such as fetal-type alkaline phosphatase, OCT4, OCT4A, SSEA-1, CXCR4, DDX4/VASA, DPPA3/STELLA, FRAGILIS/FIGLA, NOBOX, and HDAC6. These data indicated that they could be indeed developmentally related to a population of migrating epiblast-derived PGCs [43]. In addition, like other research groups before us, we were able to show that VSEL-like cells have the ability to differentiate into stem cells of three different germ layers [16, 44]. In this context it could be demonstrated that murine or human VSEL like cells were differentiable into neuron marker expressing cells *in vitro* [45] and *in vivo* [46]. However, it is not elucidated whether VSELs or other adult pluripotent stem cells like multipotent adult progenitor cells (MAPCs) [47], spore like cells (STAPs) [48] or multilineage differentiating stress enduring cells (MUSE) [49] have the capacity to differentiate into functional neurons. Here we wanted to demonstrate that the VSEL containing SCS forming cell part of the whole WJ MSC fraction is able to differentiate step wise into functional neuron like cells and could confirm that only SCS were able to express early ectodermal marker PAX6 and SOX-2 after early ectodermal induction. However, we detected also sporadically single immunofluorescence signals located in the cytoplasmic region of large adherent cells and suggest that these bright spots resulted from adhering tiny roundish cells, due to the fact that transcription factor proteins are expected in nucleus and not in cytoplasmic regions. By the reason that VSELs are quiescent under normal cell culture conditions, we can assume that they were activated through the early ectodermal pre-induction procedure, otherwise PAX-6 expression would have remained negatively regulated, as in quiescent VSELs [50].

Taking into account that we concentrated the portion of pluripotent cells through SCS formation, we expected that the probability to differentiate SCSs into active neurons is higher than in the aMSC fraction. However, due to the fact that aMSCs are still connected to a minor population of single VSEL cells, a complete exclusion of neuronal differentiation from adherent cultures is not expected. Furthermore, bigger roundish cells adhered to the surface are probably, by reason of their high nuclei to cytosol ratio, also of higher stem potency [51] which maybe also could derive from occurring and -with CD45 confirmed- (supplemental data, S1) umbilical cord blood impurities. In order to gain maximal differentiation success, our comprehensive differentiation protocol consisted of biochemical and mechanical induction agents. For mechanotransduction, we imitated the brain tissue parameters stiffness and topography [52–54], based on the fact that environmental cues of the tissue surrounding are known to be a powerful tool for stem cell development / regeneration and can be used for *in vitro* differentiation purposes [55]. To provide a neuro-inductive topography we engineered a template structured with parallel arranged micro- and nano-grooves using diamond milling and molded this structure into UV curable acrylate. Preliminary data has shown the neuro-inductive effect of this topography alone on MSCs, as an increase in both neuronal marker gene expression and protein expression could be detected (supplemental data, S5). For this study we strengthen the mechanical induction, by molding the neuro-inductive topography into GelMA with brain specific stiffness. GelMA is known to have favorable chemical and mechanical properties for potential application in traumatic brain injury [56, 57]. For initial adhesion SCS or aMSC were seeded on the semi-wet GelMA and underwent a transient FCS treatment before the biochemical neuroinduction. It has to be considered that the composition of cell types among SCSs and aMSCs is unknown, it can be assumed that only a portion of cells with concomitant plasticity are able to recognize this first mechanical induction impulse of soft biomimetic GelMA. The mechanism of this recognition remains elusive and is complicated by not only the diversity of cell types, the different maturation stages of precursor cells but also the influence to each other. Following the aim to select only neuro-potent cells, cells from SCSs or aMSCs were further treated under FCS free conditions with EGF, FGF, IGF, ß-Me-OH, N2 and B27 [58–61]. While SCS derived cells remained adhered to the GelMA a big portion of aMSC died after addition of this first induction media. However, a small portion of cells survived and changed their morphology from large flattened cells into slim cells with long extensions. Strikingly, for both outgrown SCS and aMSC cells, after this first neuro-inductive procedure, tiny (7 µm, probably VSELs), and also bigger (10-20 µm), roundish cells were still visible and did not alter their morphology in response to the differentiation stimuli. Therefore, we stimulated with a second biochemical induction step using ectodermal preinduction media. This medium has been shown in our pre-experiments to induce expression of the transcription factor PAX-6, which is a pre-condition for neuronal lineage development of pluripotent cells. In retrospect, ectodermal preinduction medium was a necessary process because experiments performed at the same time but omitting early ectodermal stimulation had lower neuronal marker expression after induction (data not shown). In order to complete the neuronal maturation process, we finally applied NT3 and cAMP [62, 63] and could observe that cell density and quantity were reduced and a lot of cells gained typical neuronal shapes.

Gene expression analysis could confirm the neuronal character and the successful mode of action of our compre-hensive neuronal induction protocol as we could demonstrate enhanced expression of RBFOX3, NEF, SCN9A, KCNB1, and NES when compared to un-induced SCS. Strikingly for aMSC, adhesion to grooved versus smooth substrates had an inhibitory effect on the expression of almost all markers. The only exception was RBFOX3 as it was up-regulated in aMSCs through groove induction. In contrast to aMSC, topography had no suppressing effect on SCS gene expression and the rates of expression significantly exceeded corresponding gene expression rates from aMSC. Also, important to note is that adhesion to grooved surface strongly enhance RBFOX3 and SCN9A1 expression and simultaneously suppressed the KCNB1 expression. In summary, we can state that grooves have a different impact on aMSC and SCS, which can be explained by a different proportion of higher potent stem cells and the different seeding techniques. Only for RBFOX3 we could demonstrate for aMSC and for SCS that mechanical cues together with biochemical neuronal induction synergistically led to expression enhancement.

From a gross comparison between aMSC and SCS we can state that, spheroid formation appears to favor the expression of neuronal markers less than suspected, as neuronal induction of aMSCs was able to achieve comparably high expression of NEFH, TUBB3, NES and SNC9A (for smooth surfaces) on average. However, SCS outgrown cells reveal the strongest expression for RBFOX3, KCNB1 and SNC9A (grooved surface). In comparison to other studies, the expression of RBFOX3 is remarkable and might stand in connection with strong SCNA and KCN expression [64]. To our knowledge, this is the first report showing such a strong RBFOX3 expression of neuronal differentiated stem cells. RbFox3 is an important nuclear factor as it regulates essential splicing events that allow the generation of multiple transcripts and isoform proteins from the same gene to promote neuronal differentiation and axon initial segment assembly during embryonic brain development [21]. *In vitro*, the translational expressed protein product of RbFox3, NeuN was often detected and interpreted as a sign of neuronally differentiated cells, but it has to take into account that MSCs from bone marrow also constitutively express NeuN as well as nestin and ß-III-tubulin [65–68]. In a further study, demonstrating the effect of photo-biomodulation on neural differentiation of UC-MSC, uninduced control samples show weak NeuN expression, which could be enhanced through induction with cerebral fluid [69].

Nevertheless, we take the detected nuclear NeuN staining seriously as we could also measure corresponding gene up-regulation. Based on our data, we demonstrated that adhesion of aMSC and SCS on grooved hydrogels significantly increased the expression of RBFOX3 genes. Accordingly, these groove topographies may be a useful tool to enhance neuronal differentiation *in vitro* in the future. This suggestion follows our hypothesis that RBFOX3 needs to reach a threshold to successfully trigger neuronal differentiation *in vitro*. The combined induction protocol achieves this threshold, as we could also measure electrophysiological activity of differentiated cells.

Calcium imaging experiments, primarily used to screen for the neuronal-like activity, have confirmed that a fraction of investigated cells express synapsin, since the genetically encoded calcium indicator was expressed exclusively under the synapsin promoter. We detected calcium signals that correspond to the neuronal-like activity in SCSs and in aMSCs. Although aMSCs show similarly high expression of some neuronal marker genes, they displayed pointedly lower active cell density than SCSs. Remarkably, around 65% of differentiated SCSs cultures showed at least some activity, which is about four times higher than previous reports on human umbilical cord blood cells [70]. Since SCSs offered a higher density of neuronal-like activity, randomized patch-clamp experiments were performed to investigate their electrical properties. Several non-linearities, detected in current- and voltage-clamp, point to neuron-like behaviors in SCSs. Firstly, we demonstrate the presence of voltage-gated ion channels. Complementary to the high expression of sodium and potassium voltage-gated ion channels, we detected currents similar to those that carry action potentials in neurons. Secondly, we registered spiking capability upon electrical stimulation. Similar to action potentials, these spikes showed all-or-none behavior and regeneration. Finally, strong hyperpolarizations induced responses similar to HCN channel activation. HCN channels, reported for neuron pacemaker cells, are essential for multispiking activity and rhythm generation. Overall, intracellular electrophysiology confirmed the potential of SCSs to differentiate into excitable, electrogenic cells with neuron-like properties.

In summary, we can say that the UC tissue contains stem cells that can be differentiated into functional neurons, especially from those cells that form spheres. Compared to the adherent cell fraction, sphere-grown cells adhered very stably to the hydrogel surface and survived harsh neuro-inductive conditions with only short-term FCS treatment. We show that only sphere forming cells show pluripotent properties. Spheres can be differentiated into early ectodermal lineages and further to electrically active neuron-like cells in the absence of mature neurons to induce them. This makes them the most promising source for neuronal cell regenerative therapies. We suspect that VSELs in combination with other sphere-forming cells are significantly involved.

However, for the use of UC tissue for the production of neuronal cell substitutes, the identification of the predestined stem cells is essential. With increasingly specific assignment of which signaling cascades are triggered by the combined application of biochemical and mechanotransductive stimuli, neuronal induction can be reduced to the essentials. Moreover, it can be elucidated why grooved topography down- or up-regulates the expression of neuronal markers in aMSCs and SCSs. Conveniently, the mechanical stimulation substrate is at the same time a scaffold and clinically applicable. When GelMA is used as an inductive substrate, differentiated neurons no longer need to be trypsinized but can be transplanted with the GelMA. This preserves the structure of the cells generated on the substrate and improves handling and reducing the chances that cells again alter their state due to detachment and relocation into the body. In addition, the imprinted grooved topography is very useful for producing tissue engineered grafts that require neurons with a specific orientation, especially in peripheral neuronal cell replacement to connect axon ends to restore conductivity. A further advantage of the proposed method for personalized medicine is that a differentiation protocol notably shorter (35 days) than that used for differentiating hPSCs into neurons (11 weeks [71]) was able to generate electrogenically active cells. Processes to directly utilize MSCs without first inducing additional pluripotency will streamline production of patient material and reduce the possibility of other undesired lineages from emerging.

## Conclusions

We demonstrated that the combined use of mechanical and chemical differentiation stimuli induced pluripotent cells from WJ to achieve a neuronal fate. In addition to gene expression and protein markers, cells with active electrogenic properties resembling functional neurons were identified after differentiation. Selection of spheroid-forming cells during the initial differentiation protocol enriches the population of cells that are functionally neuronally differentiable. Micro/nano-grooved topography had a differential effect on neuronal gene regulation in aMSC and SCS, but significantly enhanced RBFOX3 expression in both cell populations. In addition, topography also induces parallel alignment of the cells, which will be important for future applications as cell replacement therapy. To further develop and exploit the differentiation strategy developed here, it is important to elucidate underlying signaling pathways resulting from simultaneously applied mechanical and biochemical neuronal stimulation. Thus, neuronal replacement from ethically approved adult stem cells could be created under controlled conditions in the future.

## Supplementary Information

Below is the link to the electronic supplementary material.

Supplementary file1 (JPG 273 KB) Figure S1 Flow cytometry analysis of adherent WJ-derived cells. The surface antigen expression was measured for CD73, CD90, CD105, as well as for the negative marker mixture comprising CD14, CD20, CD34 and CD45. The rows represent the antigen expression measurements of cells that have been cultivated on culture vessels. The surface antigen expressions (blue peak) were determined with respect to appropriate isotype controls (red peak). Signals exceeding a fluorescence intensity of 10^3^ were interpreted as positive. The image shows one representative example of flow cytometry analysis of three independent donors

Supplementary file2 (JPG 133 KB) Figure S2 Expression of pluripotency marker in SCS: Comparison of free-floating spheroids against adherent spheroids. Relative normalized gene expression of SOX-2, POU5F1 and NANOG of Jurkat cells (negative control) against adherent UC-MSC derived spheroids and free-floating UC-MSC derived spheroids. The gene expression data were normalized by means of the housekeeping genes GAPDH and B2M. The error bars indicate the standard deviation for three technical replicates

Supplementary file3 (JPG 105 KB) Figure S3 Isotype Control of protein expression analysis of Nanog, Sox2 and SSEA4 staining of WJ-MSC. Isotype control staining of Nanog/Sox2, Oct-4 or SSEA-4 antibodies (red). The nuclei were stained with DAPI (blue). Scale bars represent 50 µm

Supplementary file4 (JPG 630 KB) Figure S4 Protein expression of pluripotency marker in spheroid resident cells: single cell stainings are visible. Protein expression analysis of Nanog, Sox-2, Oct-4 in SCSs with a 200xfold magnification make immunostaining of single cells visible. a) co-staining of Sox-2 and Nanog. b) Isotype controls of a. c) Oct-4 staining of spheroid cells d) Isotype control of c. The nuclei were stained with DAPI. Scale bars represent 50 µm

Supplementary file5 (JPG 238 KB) Figure S5 Effect of topography on MSCs on neuronal marker expression. A: Micro-structured Polydimethylsiloxane (PDMS) as mechanotransductive substrate. Left top: SEM of PDMS mold with structure 1 (a, left) and structure 2 (c, right) , imaged at 1000 fold magnification and bottom: structure 1 (b, left) and structure 2 (d, right) at 5000 fold magnification. B: RT PCR analysis of adipose derived stem cells (ADSCs) to analyze the induction of neuronal marker gene expression after adhesion to micro-structured PDMS surfaces. ADSC in passage 3, cultivated on S1 and S2 micro-structured PDMS and on flat PDMS (control) to investigate the gene expression of NES, NEFH and TUBBIII. Gene expression data were normalized by means of the housekeeping genes GAPDH, RPLO and 18s rRNA. Error bars indicate the standard deviation of three technical replicates. C: Immunofluorescence analysis of ADSC in passage 3, cultivated on flat, S1 or S2 micro-structured PDMS surfaces. Red fluorescence represents the expression of Nestin and ß-Tubulin. Nuclei stained with DAPI shown in blue. Scale bars represent 75µm

Supplementary file6 (JPG 126 KB) Figure S6 Expression of TMEM119, DCX and NEUROD1. Relative normalized gene expression of TMEM, DCX and NEUROD1 in Jurkat cells (as negative control) against differentiated SCS cultured on smooth (a and b) or micro/nano grooved GelMA (c and d) hydrogels for 35 days. The gene expression data were normalized by means of the housekeeping genes GAPDH and B2M. The error bars indicate the standard deviation for three technical replicates

## Data Availability

All authors ensure that all data and materials support the published claims and comply with field standards.

## Declarations

### Patents

US2021115393A1 “Neuronally inductive cultivation matrix” Inventor: Angela Gutermuth, Christoph Baum.
