# Supplementary figures and images for: In Vitro Simulated Neuronal Environmental Conditions Qualify Umbilical Cord Derived Highly Potent Stem Cells for Neuronal Differentiation
Source: Stem Cell Rev Rep. 2023 Apr 24;19(6):1870–89. doi: 10.1007/s12015-023-10538-w (PMC10390376; doi:10.1007/s12015-023-10538-w)

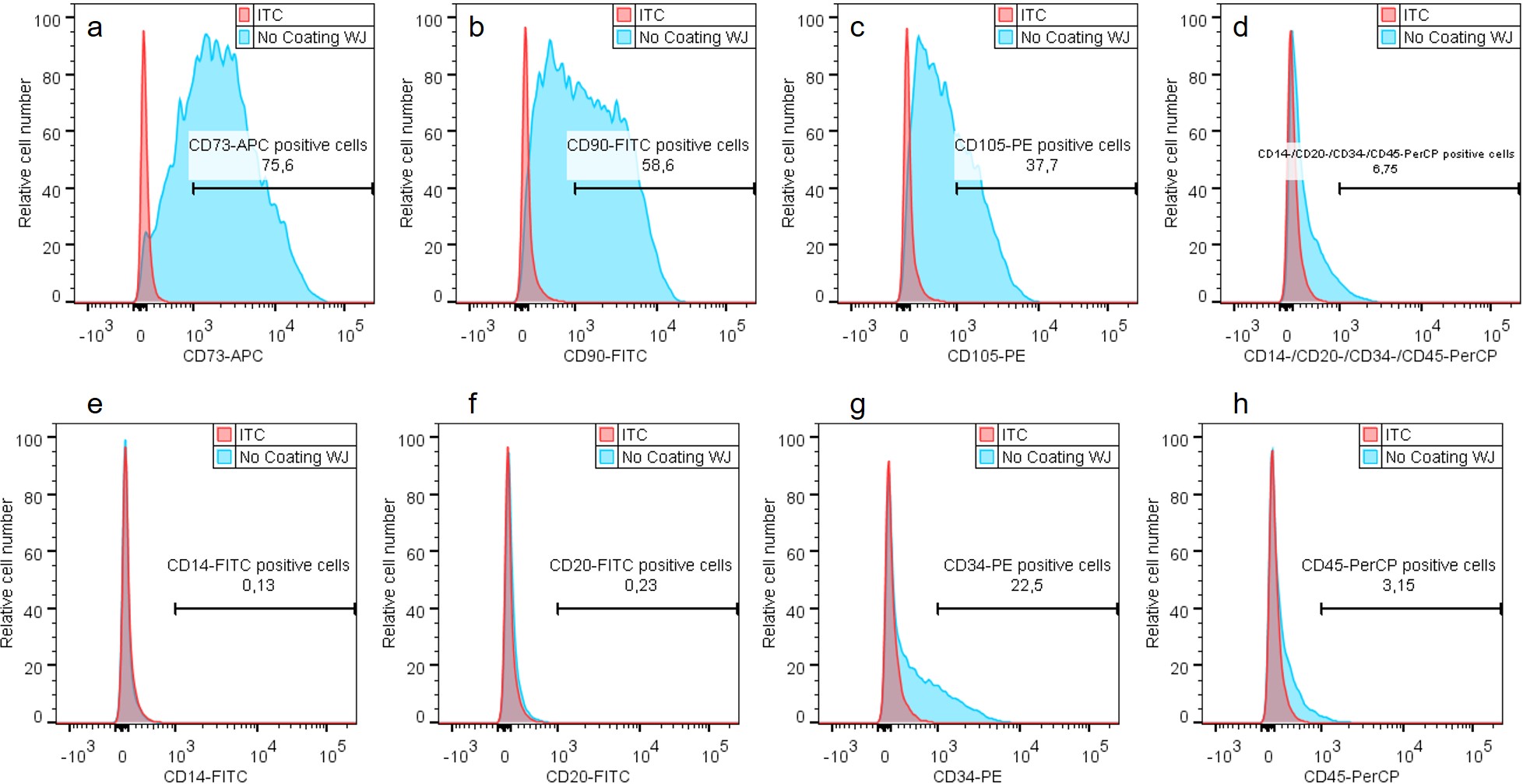

Supplement: Supplementary file 1 — Supplementary file1 (JPG 273 KB) Figure S1 Flow cytometry analysis of adherent WJ-derived cells. The surface antigen expression was measured for CD73, CD90, CD105, as well as for the negative marker mixture comprising CD14, CD20, CD34 and CD45. The rows represent the antigen expression measurements of cells that have been cultivated on culture vessels. The surface antigen expressions (blue peak) were determined with respect to appropriate isotype controls (red peak). Signals exceeding a fluorescence intensity of 103 were interpreted as positive. The image shows one representative example of flow cytometry analysis of three independent donors [file 12015_2023_10538_MOESM1_ESM.jpg]

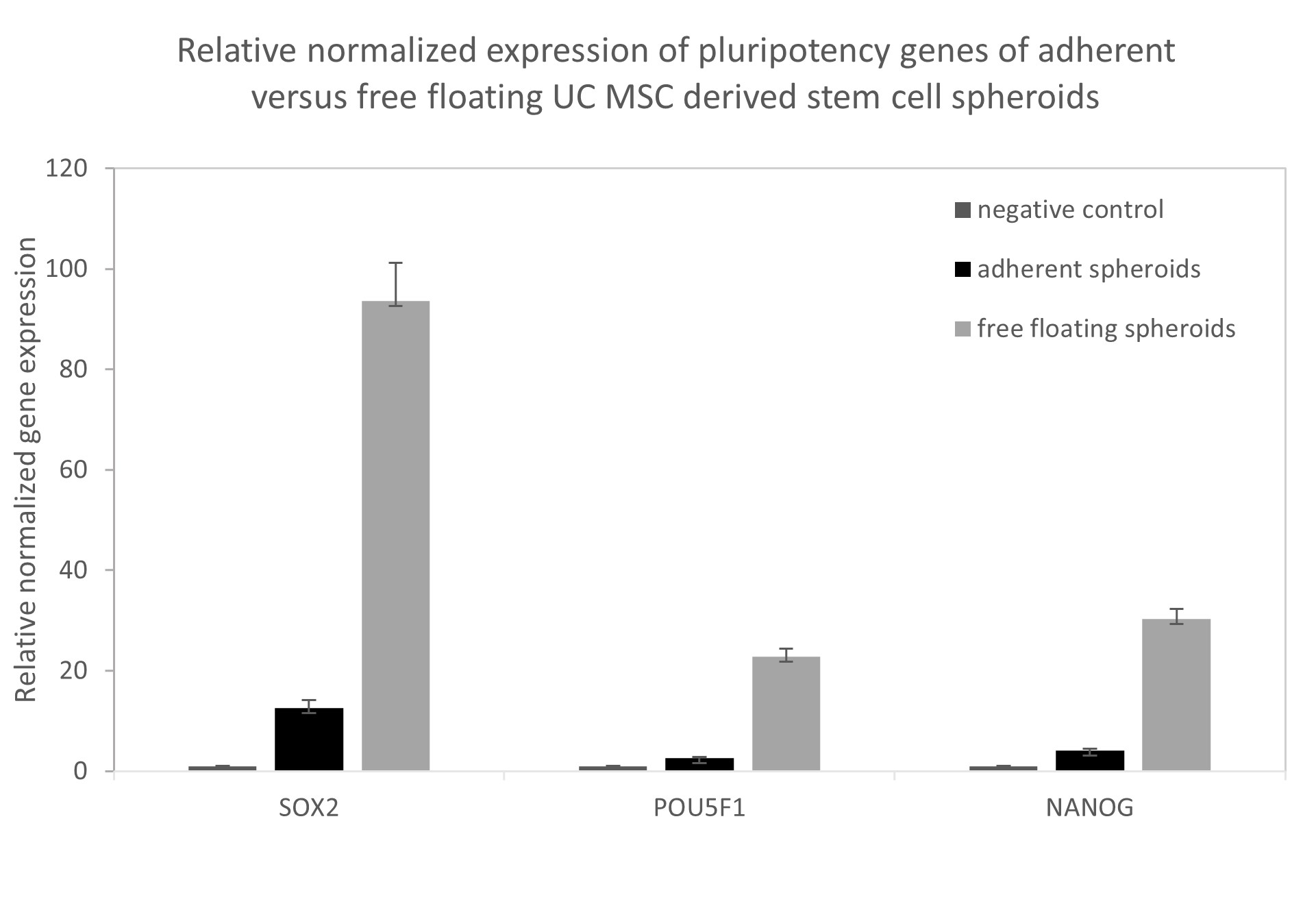

Supplement: Supplementary file 2 — Supplementary file2 (JPG 133 KB) Figure S2 Expression of pluripotency marker in SCS: Comparison of free-floating spheroids against adherent spheroids. Relative normalized gene expression of SOX-2, POU5F1 and NANOG of Jurkat cells (negative control) against adherent UC-MSC derived spheroids and free-floating UC-MSC derived spheroids. The gene expression data were normalized by means of the housekeeping genes GAPDH and B2M. The error bars indicate the standard deviation for three technical replicates [file 12015_2023_10538_MOESM2_ESM.jpg]

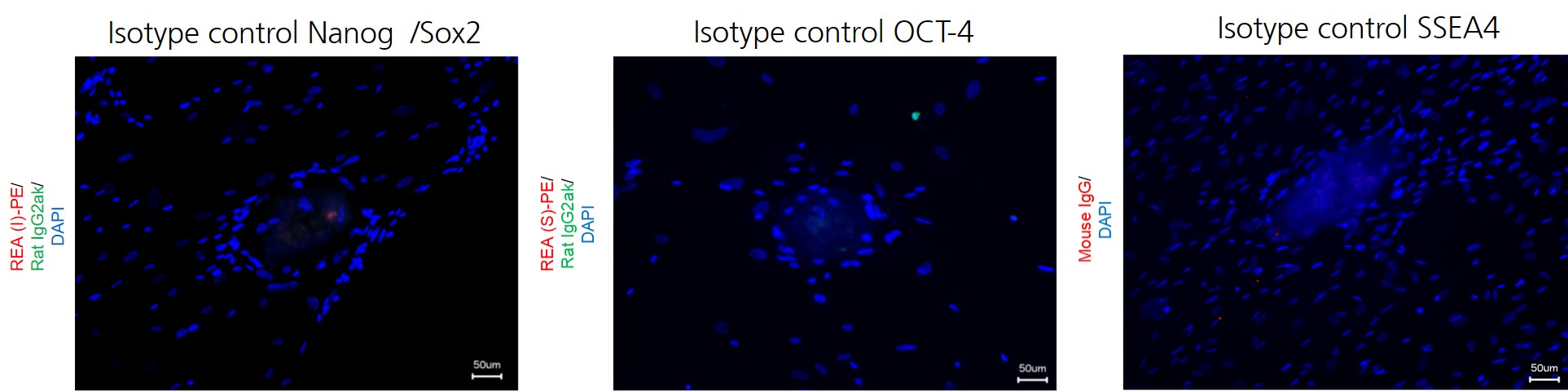

Supplement: Supplementary file 3 — Supplementary file3 (JPG 105 KB) Figure S3 Isotype Control of protein expression analysis of Nanog, Sox2 and SSEA4 staining of WJ-MSC. Isotype control staining of Nanog/Sox2, Oct-4 or SSEA-4 antibodies (red). The nuclei were stained with DAPI (blue). Scale bars represent 50 µm [file 12015_2023_10538_MOESM3_ESM.jpg]

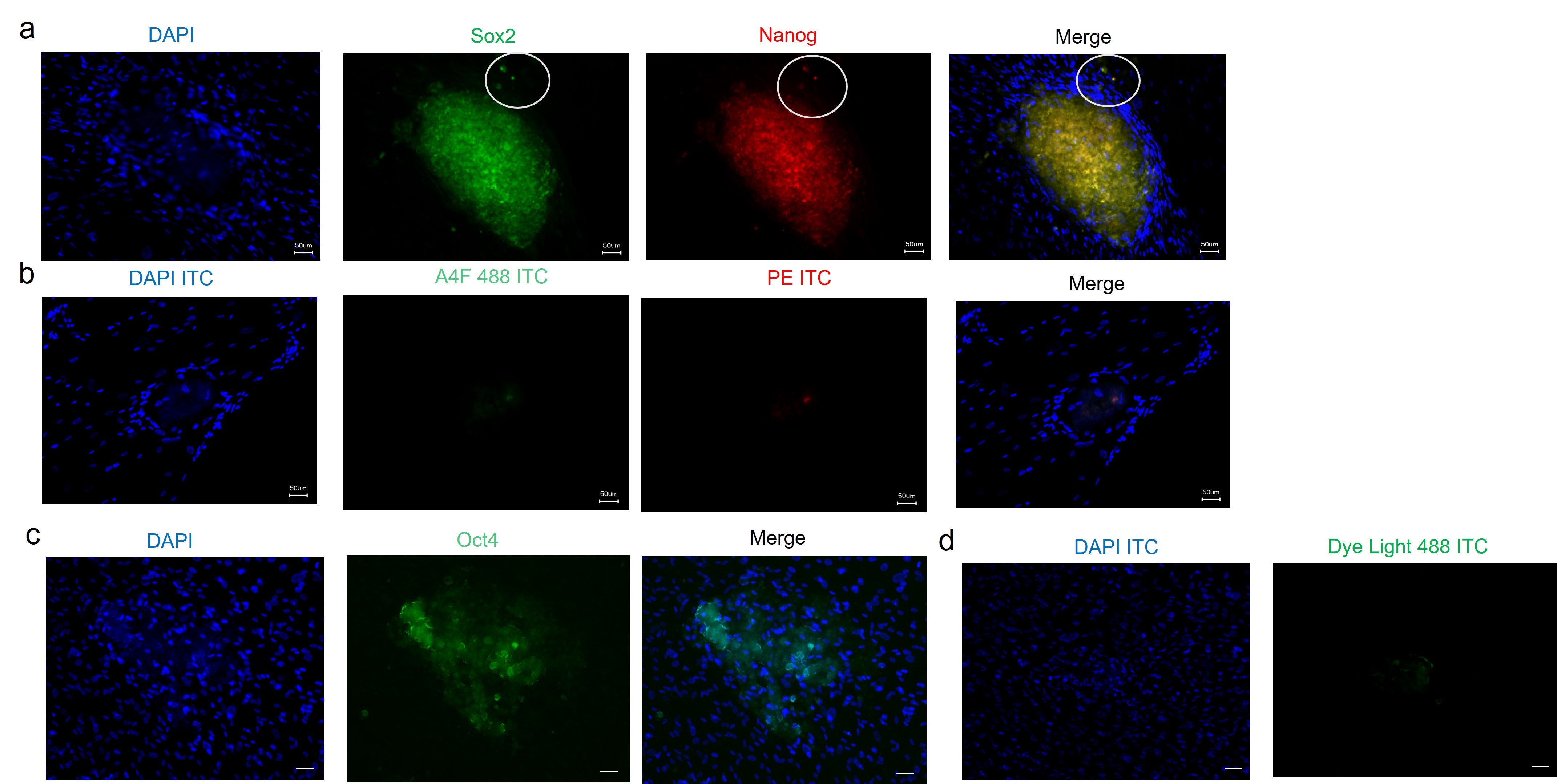

Supplement: Supplementary file 4 — Supplementary file4 (JPG 630 KB) Figure S4 Protein expression of pluripotency marker in spheroid resident cells: single cell stainings are visible. Protein expression analysis of Nanog, Sox-2, Oct-4 in SCSs with a 200xfold magnification make immunostaining of single cells visible. a) co-staining of Sox-2 and Nanog. b) Isotype controls of a. c) Oct-4 staining of spheroid cells d) Isotype control of c. The nuclei were stained with DAPI. Scale bars represent 50 µm [file 12015_2023_10538_MOESM4_ESM.jpg]

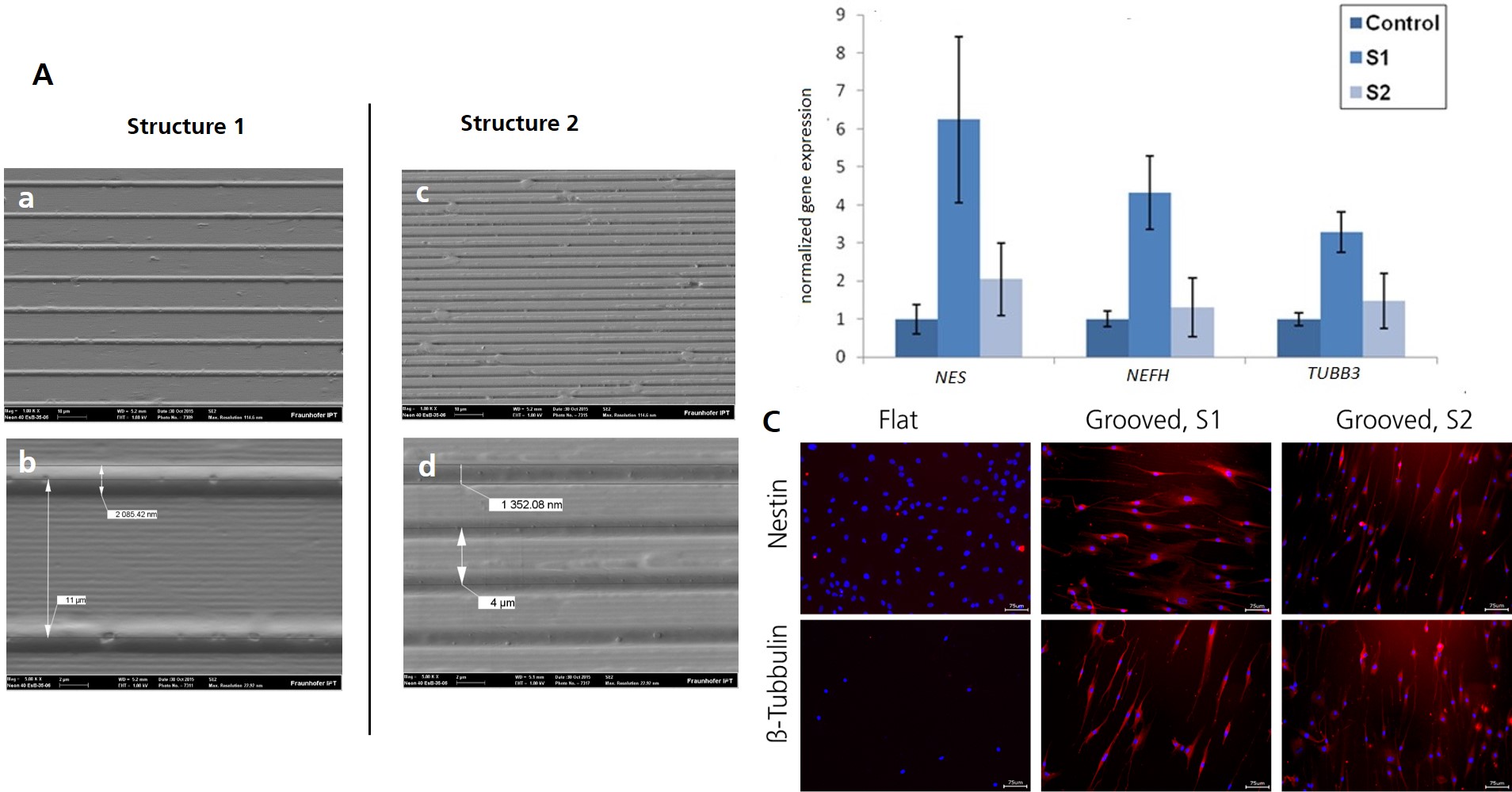

Supplement: Supplementary file 5 — Supplementary file5 (JPG 238 KB) Figure S5 Effect of topography on MSCs on neuronal marker expression. A: Micro-structured Polydimethylsiloxane (PDMS) as mechanotransductive substrate. Left top: SEM of PDMS mold with structure 1 (a, left) and structure 2 (c, right) , imaged at 1000 fold magnification and bottom: structure 1 (b, left) and structure 2 (d, right) at 5000 fold magnification. B: RT PCR analysis of adipose derived stem cells (ADSCs) to analyze the induction of neuronal marker gene expression after adhesion to micro-structured PDMS surfaces. ADSC in passage 3, cultivated on S1 and S2 micro-structured PDMS and on flat PDMS (control) to investigate the gene expression of NES, NEFH and TUBBIII. Gene expression data were normalized by means of the housekeeping genes GAPDH, RPLO and 18s rRNA. Error bars indicate the standard deviation of three technical replicates. C: Immunofluorescence analysis of ADSC in passage 3, cultivated on flat, S1 or S2 micro-structured PDMS surfaces. Red fluorescence represents the expression of Nestin and ß-Tubulin. Nuclei stained with DAPI shown in blue. Scale bars represent 75µm [file 12015_2023_10538_MOESM5_ESM.jpg]

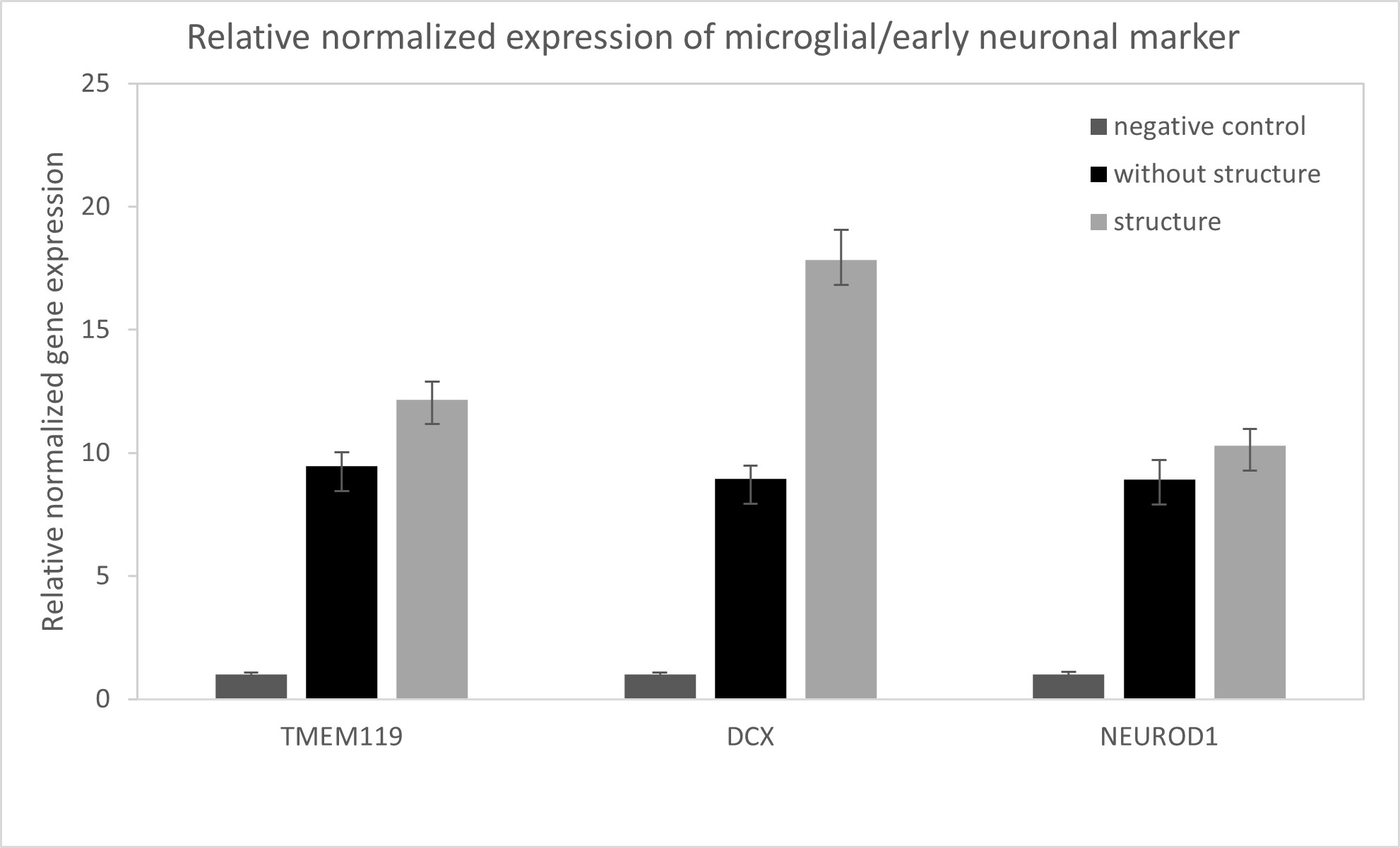

Supplement: Supplementary file 6 — Supplementary file6 (JPG 126 KB) Figure S6 Expression of TMEM119, DCX and NEUROD1. Relative normalized gene expression of TMEM, DCX and NEUROD1 in Jurkat cells (as negative control) against differentiated SCS cultured on smooth (a and b) or micro/nano grooved GelMA (c and d) hydrogels for 35 days. The gene expression data were normalized by means of the housekeeping genes GAPDH and B2M. The error bars indicate the standard deviation for three technical replicates [file 12015_2023_10538_MOESM6_ESM.jpg]
